# A Control Scheme That Uses Dynamic Postural Synergies to Coordinate a Hybrid Walking Neuroprosthesis: Theory and Experiments

**DOI:** 10.3389/fnins.2018.00159

**Published:** 2018-04-10

**Authors:** Naji A. Alibeji, Vahidreza Molazadeh, Brad E. Dicianno, Nitin Sharma

**Affiliations:** ^1^Department of Biomedical Engineering, Case Western Reserve University, Cleveland, OH, United States; ^2^Department of Mechanical Engineering and Materials Science, University of Pittsburgh, Pittsburgh, PA, United States; ^3^Department of Bioengineering, University of Pittsburgh, Pittsburgh, PA, United States

**Keywords:** hybrid neuroprosthesis, synergy, nonlinear control, neuromuscular stimulation, Lyapunov

## Abstract

A hybrid walking neuroprosthesis that combines functional electrical stimulation (FES) with a powered lower limb exoskeleton can be used to restore walking in persons with paraplegia. It provides therapeutic benefits of FES and torque reliability of the powered exoskeleton. Moreover, by harnessing metabolic power of muscles via FES, the hybrid combination has a potential to lower power consumption and reduce actuator size in the powered exoskeleton. Its control design, however, must overcome the challenges of actuator redundancy due to the combined use of FES and electric motor. Further, dynamic disturbances such as electromechanical delay (EMD) and muscle fatigue must be considered during the control design process. This ensures stability and control performance despite disparate dynamics of FES and electric motor. In this paper, a general framework to coordinate FES of multiple gait-governing muscles with electric motors is presented. A muscle synergy-inspired control framework is used to derive the controller and is motivated mainly to address the actuator redundancy issue. Dynamic postural synergies between FES of the muscles and the electric motors were artificially generated through optimizations and result in key dynamic postures when activated. These synergies were used in the feedforward path of the control system. A dynamic surface control technique, modified with a delay compensation term, is used as the feedback controller to address model uncertainty, the cascaded muscle activation dynamics, and EMD. To address muscle fatigue, the stimulation levels in the feedforward path were gradually increased based on a model-based fatigue estimate. A Lyapunov-based stability approach was used to derive the controller and guarantee its stability. The synergy-based controller was demonstrated experimentally on an able-bodied subject and person with an incomplete spinal cord injury.

## 1. Introduction

Paraplegia in persons with spinal cord injury (SCI) impairs walking function and lowers their quality of life. Functional electrical stimulation (FES) and powered exoskeletons are two potential technologies that aim to reanimate lower-limb function in these persons. FES is an artificial application of electrical potential across a muscle group to produce a desired limb function and is prescribed as an intervention to rehabilitate or restore gait function in individuals with mobility-impairements (Peckham and Gray, 1996). FES was used for the first time in the 1960s by Kantrowitz (1960) and Liberson et al. (1961) to produce gait patterns and to correct drop foot, respectively. Since then FES systems that use either percutaneous or surface electrodes have been used to produce gait (Bajd et al., 1983; Marsolais and Kobetic, 1987; Kralj and Bajd, 1989; Granat et al., 1993; Kobetic et al., 1997; Hardin et al., 2007). Despite this progress, the issue of rapid onset of FES-induced muscle fatigue remains unresolved. To reduce the effects of muscle fatigue, FES has been used in conjunction with a passive orthosis (Solomonow et al., 1988; Goldfarb et al., 2003; Farris et al., 2009; Kobetic et al., 2009). The addition of an orthosis mitigates fatigue effects by lowering stimulation duty cycle of FES because it can be used to support the user's weight during standing. However, the gait is still powered by FES during the swing movement and is affected by FES-induced muscle fatigue.

Powered exoskeletons by their virtue of generating high, rapid, and reliable torque are actively being used to provide gait therapy or restoration (Farris et al., 2011; Neuhaus et al., 2011; Strausser and Kazerooni, 2011). Compared to sole FES-based walking systems, however, they may have higher power consumption to operate high torque motors. Bulky high torque motors and larger batteries increase weight and reduce wearability. A hybrid device that combines an FES system with a powered exoskeleton (del Ama et al., 2012, 2014; Ha et al., 2012; Kirsch et al., 2013, 2014a) can overcome these limitations by reducing power consumption and actuator size in the powered exoskeleton. Moreover, the use of FES provides therapeutic benefits to a user.

In Quintero et al. (2012), FES was combined with a powered exoskeleton to control knee extension by using an adaptive gain-based controller and a PD controller. In del Ama et al. (2014) a cooperative knee joint controller was used in a hybrid knee-ankle-foot exoskeleton. The approach was tested on able-bodied subjects. A PID controller and an iterative learning controller were used to stimulate the quadriceps muscle and the knee flexors, respectively while a variable stiffness controller computed the knee electric motor stiffness based on the measured interaction torque between the user and the exoskeleton. In Ha et al. (2015), another cooperative control approach was used to coordinate hip motors with the stimulation of the hamstrings and knee motors with the stimulation of quadriceps muscle. The approach was tested on three subjects with SCI. The motors were controlled using a high-bandwidth position feedback and the FES control was modified by the difference between the estimated muscle torque and the reference torque profile.

In our previous research, a dynamic optimization method was used to optimize a hybrid walking system (FES + passive orthosis) (Sharma et al., 2014). However, the method computes FES control inputs offline. Motivated to develop an optimization method for a real-time implementation, in Kirsch et al. (2014b), a linear model predictive control (MPC) method was proposed to dynamically allocate control in a hybrid knee joint control system composed of FES and an electric motor. However, a linearized musculoskeletal model was used for the linear MPC method, which may lose control performance outside the region of linearization. Therefore a nonlinear model predictive controller (NMPC) for an FES only case was developed in Kirsch and Sharma (2017) to elicit knee extension in able-bodied participants.

Aforementioned research papers in hybrid neuroprosthesis control focused primarily on coordinating FES and the motors at a single joint, even though some of these papers provided pioneering evidence of its benefits. Motivated to provide a general framework that coordinates stimulation of multiple muscles and exoskeleton actuators at multiple joints, a muscle synergy-inspired controllers were presented in Alibeji et al. (2015b, 2017). In Alibeji et al. (2015b), simulations of the synergy inspired controller for single stepping motion were shown. This controller was further improved to incorporate effects of fatigue and electromechanical delay (EMD) in Alibeji et al. (2017). The experimental evidence of the synergy-inspired controller was provided using standing-cyclical experiments.

Motivated to extend the synergy-based controller, in this paper, dynamic postural synergies were used in a control scheme to generate walking with a hybrid exoskeleton. The dynamic postural synergies are artificial synergies designed to drive the system to key dynamic postures when activated. Then sequential activation of these dynamic postural synergies drive the system to produce gait motions. An adaptive update law was used to modify the synergy activation profiles to compensate for parametric changes in the model. A PID-based feedback component was used to make the controller robust to uncertainity and disturbances. The controller uses dynamic surface control (DSC) (Alibeji et al., 2017) to avoid the use of acceleration signals in the control design. This DSC framework was also modified to include a delay compensation term to account for the EMD. To counter muscle fatigue effects, the control input terms were scaled by the fatigue estimate's inverse. In addition, a scaling factor gain is added to the feedforward component in case there is mismatch in model and subjects strength during experiments. Model-based estimators were designed to estimate the fatigue and activation state variables. The individual components of this controller have been validated experimentally and through simulations in Sharma et al. and Alibeji et al. and have been shown to provide improved performance compared with traditional PID controllers (Sharma et al., 2011; Alibeji et al., 2015a,b, 2017). Finally, experiments were performed on an able-bodied subject and a person with an incomplete spinal cord injury to show the feasibility of coordinating multiple muscles and electric motors with the synergy-inspired controller.

## 2. Methods

### 2.1. Walking hybrid neuroprosthesis model

**Figure 1:**
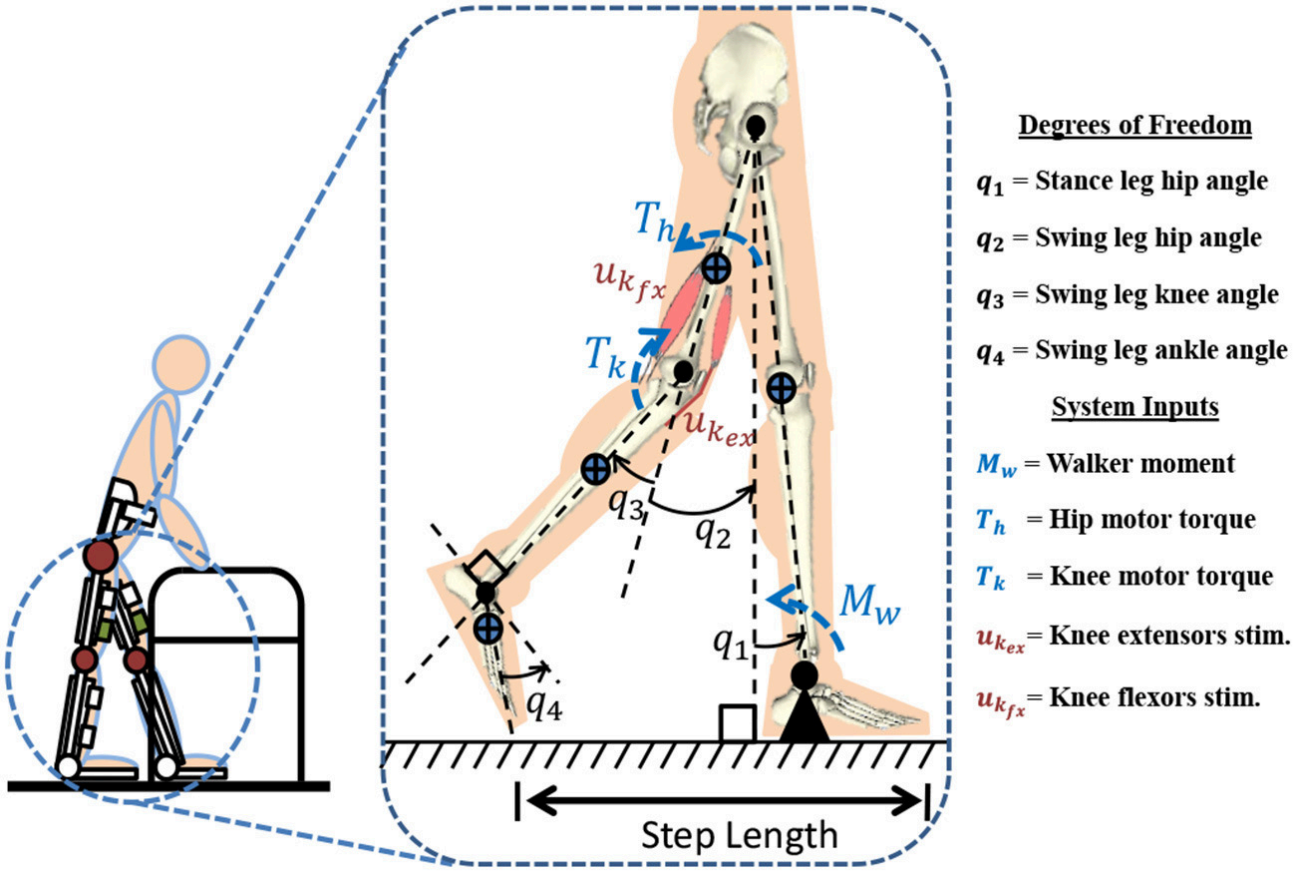
A 4-link gait model is used to represent a subject taking a step in a hybrid neuroprosthesis while using a walker.

Figure 1 represents the 4-link model which is used for modeling a hybrid neuroprosthesis and a walker. The 4-link model considers a hybrid neuroprosthesis that uses electric motors and FES via surface electrodes, which non-selectively apply an external voltage potential to a muscle group to generate a contraction. The stance leg is modeled as one rigid segment simulating the locking of the knee joint and the ankle is fixed to the ground because only half of the gait cycle is considered. The swing leg has a thigh, shank, and foot segment but only the hip and knee joints have active actuation. The knee joint uses 3 actuators: motor and FES for flexion and extension of antagonistic muscle pairs. The model only uses electric motors at the hip joints because it can be difficult to stimulate the hip flexors and extensors, as these muscle are not easily accessible using surface electrodes. The trunk dynamics were neglected in the model because the use of a walker allows the user to stabilize their truck. However, the model assumes the trunk is fixed at the vertical orientation. The walker is modeled as a moment acting on the stance leg to help propel the body forward and also to keep it upright. The lower limb model is given as:

(1) $$\begin{array}{l} M\left(q\right)\ddot{q}+C\left(q,\dot{q}\right)\dot{q}+G\left(q\right)+f\left(q,\dot{q}\right)+\Gamma_{d}\left(t\right)+\Gamma_{ext}\left(t\right)=\Gamma , \end{array}$$

where $q,\dot{q},\ddot{q}\in {\mathbb{R}}^{4}$ are the angular positions, velocities, and accelerations of the leg segments, respectively. In (1), *M*(*q*) ∈ ℝ^4×4^ is the combined inertia of the hybrid neuroprosthesis and human limbs, $C\left(q,\dot{q}\right)\in {\mathbb{R}}^{4\times 4}$ is the centripetal/Coriolis matrix, *G*(*q*) ∈ ℝ^4^ is the gravity vector, $f\left(q,\dot{q}\right)\in {\mathbb{R}}^{4}$ is the viscoelastic vector term that models the passive muscle dynamics, $\Gamma_{ext}\in {\mathbb{R}}^{4}$ is the torque generated at each joint due to contact with the ground and walker moment (*M*_*W*_), and $\Gamma_{d}\in {\mathbb{R}}^{4}$ is any unmodeled effects or disturbances in the system. The active torques at the joints are generated by including the musculoskeletal dynamics due to FES (Popović et al., 1999), an electric motor attached at each joint, and the moment generated by the walker force. The torque term is defined as

(2) $$\begin{array}{l} \Gamma =b\left(q,\dot{q}\right)\phi \left(t\right)\mu \left(t\right), \end{array}$$

where μ(*t*) ∈ ℝ^4^ is the intermediate normalized activation vector containing activation states for the actuators, and is defined as

$$\mu ≜{\left[\begin{array}{cccc} \mu_{k_{fx}} & \mu_{k_{ex}} & \mu_{h_{m}} & \mu_{k_{m}} \end{array}\right]}^{\text{T}},$$

where, μ_*k*_*fx*__ ∈ ℝ is knee flexor muscle activation, μ_*k*_*ex*__ ∈ ℝ is knee extensor muscle activation, μ_*k*_*m*__ ∈ ℝ is normalized current for the knee motor and μ_*h*_*m*__ ∈ ℝ is normalized current for the hip motor. In (2), ϕ(*t*) ∈ ℝ^4×4^ is the fatigue matrix that contains the fatigue factor corresponding to each stimulated muscle and is defined as

$$\phi ≜diag\left(\left[\begin{array}{cccc} \phi_{k_{fx}} & \phi_{k_{ex}} & 1 & 1 \end{array}\right]\right),$$

and $b\left(q,\dot{q}\right)\in {\mathbb{R}}^{4\times 4}$ is the control gain matrix defined as

(3) $$\begin{array}{l} b={\left[\begin{array}{cccc} 0 & 0 & \psi_{k_{fx}} & 0\\ 0 & 0 & -\psi_{k_{ex}} & 0\\ 0 & \kappa_{h} & 0 & 0\\ 0 & 0 & \kappa_{k} & 0 \end{array}\right]}^{T}, \end{array}$$

In (3), ψ_*i*_*fx*__, ψ_*i*_*ex*__ are the torque-length and torque-velocity relationships of the flexor and extensor muscles and the conversion constants (current to torque) of the electric-motor drives is κ_*i*_.

The activation state is governed by the following first order differential equation

(4) $$\begin{array}{l} {\dot{\mu }}_{i_{j}}=-\omega_{i_{j}}\mu_{i_{j}}+\omega_{i_{j}}u_{i_{j}}\left(t-\tau_{i_{j}}\right), \end{array}$$

where subscripts *i* = *h, k* stand for the hip and knee joints of the swing leg and (*j* = *fx, ex, m*) for the type of actuator. In (4), $\omega_{i_{j}}\in {\mathbb{R}}^{+}$ is the actuator decay constant, *u*_*i*_*j*__ is the normalized input, and τ_*i*_*j*__ is the input delay.

The fatigue dynamics of the muscles, ϕ_*i*_*j*__ ∈ ℝ is generated from the first order differential equation (Riener et al., 1996)

(5) $$\begin{array}{l} {\dot{\phi }}_{i_{j}}=\frac{1}{T_{f_{i_{j}}}}\left(\phi_{\min_{i_{j}}}-\phi_{i_{j}}\right)\mu_{i_{j}}+\frac{1}{T_{r_{i_{j}}}}\left(1-\phi_{i_{j}}\right)\left(1-\mu_{i_{j}}\right), \end{array}$$

where ϕ_min_ ∈ (0, 1) is the unknown minimum fatigue constant of a muscle, and *T*_*f*_, $T_{r}\in {\mathbb{R}}^{+}$ are unknown time constants for fatigue and recovery in the muscle, respectively. Because μ ∈ [*u*_*min*_, *u*_*max*_] for muscles, it can be shown that ϕ ∈ [ϕ_min_, 1], where ϕ = 1 when the muscle is fully rested, and ϕ = ϕ_min_ when the muscle is fully fatigued. The fatigue state for the motors in the fatigue matrix are set to one because the motors do not fatigue.

The stimulation applied to the muscle is bounded by two stimulation levels *v*_*min*_ and *v*_*max*_ to avoid under/over stimulating the muscles. This allows the normalization of the input function *u*(*t*) ∈ ℝ^4^, which is modeled by a piecewise linear recruitment curve (Schauer et al., 2005), as

(6) $$\begin{array}{l} u\left(t\right)=sat\left[v\left(t\right)\right]=\left\{ \begin{array}{cc} 0\; & v<v_{min}\\ \frac{v\left(t\right)-v_{min}}{v_{max}-v_{min}}\; & v_{min}\leq v\leq v_{max}\\ 1\; & v>v_{max} \end{array}\right. \end{array}$$

where $v_{\min},v_{\text{max}}\in {\mathbb{R}}^{4}$ are the minimum/maximum input magnitudes for each actuator (stimulation or motor) and *v*(*t*) ∈ ℝ^4^ is the input to the system. Based on (4) and (6), a linear differential inequality can be developed to show that μ ∈ [*u*_min_, *u*_max_]. The *u*_min_, *u*_max_ values are [0, 1] for muscles because they are unidirectional and [−1, 1] for electric motors because they are bidirectional actuators.

### 2.2. Dynamic postural synergies

The purpose of muscle synergies in human motor control is to reduce the complexity of the system by reducing the input space and redundant DOF. In this paper, an alternative form of synergies called dynamic postural synergies are introduced. Unlike other methods which identify synergies by using statistical analysis tools on collected EMG data or simulation results, this form of synergies is computed independently to create a reduced input space for a system that can be used to more efficiently control a system. The dynamic postural synergies generated in this paper are artificial synergies that are designed to drive the system to key dynamic postures, which are defined as the joint positions at any moment during a movement pattern. Then motions such as walking can be segmented into a finite number of dynamic postures and a dynamic postural synergy can be computed for each dynamic posture. These artificial synergies can then be activated sequentially to drive the system from one dynamic posture to the next to create the original motion.

In Bajd et al. (1983) rudimentary gait was recreated in subjects with SCI by stimulating the peroneal nerve and then the quadriceps to produce two key dynamic postures; the withdrawal reflex and knee extension. The withdrawal reflex is a spinal reflex that protects the body from damaging stimuli and can be triggered by activating the pain receptors at the bottom of the foot or stimulating the peroneal nerve. The reflex consists of the flexing of the hip, knee, and ankle joints to immediately lift the leg off of the ground or the source of the pain. In this work, the artificial synergies, defined as *W* ∈ ℝ^4×2^, that produce these dynamic postures were computed using dynamic optimizations. Then, another set of dynamic optimizations were used to find the optimal activation of these artificial synergies, defined as $c_{d}\in {\mathbb{R}}^{2}$, to reproduce gait trajectories, *q*_*d*_. Below, the dynamics, excluding the fatigue factor ϕ, are written in terms of the kinematic trajectories (*q*_*d*_) and the activation state generated from the dynamic postural synergies and their optimal activation (i.e., μ_*d*_ = *Wc*_*d*_) as

(7) $$\begin{array}{l} M\left(q_{d}\right){\ddot{q}}_{d}+C\left(q_{d},\dot{q_{d}}\right)\dot{q_{d}}+G\left(q_{d}\right)+f\left(q_{d},\dot{q_{d}}\right)\\ \equiv b\left(q_{d},{\dot{q}}_{d}\right)\mu_{d}\left(t\right)-\Gamma_{ext}^{*}, \end{array}$$

where $\Gamma_{ext}^{*}$ is the ground reaction forces and walker moment, *M*_*W*_, resulting from the optimal trajectories (*q*_*d*_).

#### 2.2.1. Computing the synergies

**Figure 2:**
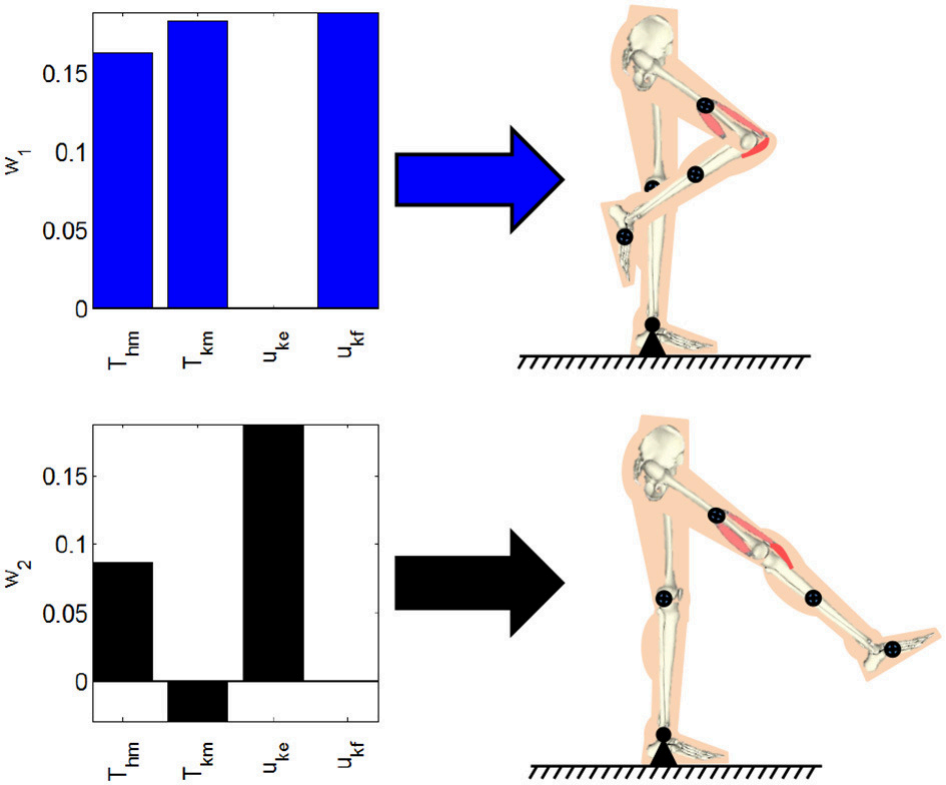
The dynamic postural synergies computed through the optimizations and the dynamic postures they result in when activated.

The dynamic postural synergies are computed using optimizations that use the 4-link walking model in (1). The 4-link walking model was modified to reflect the hybrid neuroprosthesis testbed, therefore, only the hip motors, knee motors, and the antagonistic muscle pairs of the knee joint are used. The parameters used for this model were taken from Popović et al. (1999) for an able bodied person. Optimizations were conducted to compute the synergies that distribute the effort to the 4 inputs that minimize the error between the desired dynamic posture and the resulting motion. The joint angles for the desired dynamic postures were taken from the optimal trajectories in Alibeji et al. (2015b). For these optimizations, the convex cost function's objective was to minimize the dynamic posture's position error and minimize the activation states of the system and is defined as

(8) $$\begin{array}{l} \underset{W}{\text{min}}\Pi =\overset{t_{f}}{\underset{t_{0}}{\int }}\left(E_{1}{\left(t\right)}^{T}Q_{1}E_{1}\left(t\right)+\mu {\left(t\right)}^{T}R_{1}\mu \left(t\right)\right)dt \end{array}$$

$$\begin{array}{l} \text{subject to}:M\left(q\right)\ddot{q}+C\left(q,\dot{q}\right)\dot{q}+G\left(q\right)+f\left(q,\dot{q}\right)\\ =b\left(q,\dot{q}\right)\mu -\Gamma_{ext},\\ \mu \in \left[\mu_{l},\mu_{u}\right] \end{array}$$

where dynamic posture's position error is defined as *E*_1_ = *q*_*dp*_ − *q* and *q*_*dp*_ is the joint positions for the desired dynamic posture. In (8), $Q_{1}\in {\mathbb{R}}^{4\times 4}$ is a weight on the position tracking error, the matrix $R_{1}\in {\mathbb{R}}^{4\times 4}$ is a positive-definite matrix of weights on the activation vector, and the lower and upper bound on the activations are defined as μ_*l*_ and $\mu_{u}\in {\mathbb{R}}^{4}$. Based on the selection of the input weight matrix *R*_1_, the distribution of the effort from the motors or stimulation can be emphasized. These optimizations were performed by using Matlab's fmincon function (MathWorks, Inc., USA). The dynamic postural synergies computed through the optimization and the postures they produce; withdrawal reflex and knee extension, can be seen in Figure 2. The first dynamic postural synergy activates the hip motor to produce a moment at the hip in the flexion direction, and activates the knee motor and knee flexor to produce a moment at the knee in the flexion direction, to produce the withdrawal reflex. The second dynamic postural synergy activates the hip motor to produce a smaller moment at that hip to maintain the hip joint's position, and activates the knee motor and knee extensor to produce a moment at the knee to fully extend the knee joint.

#### 2.2.2. Computing the synergies' activation

Unlike the synergies extracted through statistical methods, such as principal component analysis in Alibeji et al. (2015b), these dynamic postural synergies were determined using separate optimizations prior to these dynamic optimizations. Using these already computed dynamic postural synergies, these dynamic optimizations now compute the optimal synergies' activations in order to complete a step.

In order to consistently and easily maintain the initial condition during experimentation, the subject will start the gait process while standing upright. Therefore, two sets of dynamic optimizations are computed; one for a half step (0.2 meters) and the second for a full step (0.4 meters).

These dynamic optimizations also include the double support phase (DSP) part of the gait sequence, i.e, when the body is supported by both legs. During the DSP the load transfers from the stance leg to the swing leg and the legs switch roles, i.e., the stance leg from the previous step becomes the swing leg for the next step and vice versa. To include the DSP, the swing leg has to the reach the desired position, where the swing leg makes contact with the ground, in the allotted time, *t*_*step*_ = 1 s., and maintain that position, i.e., maintain contact with the ground, for a predetermined duration, *t*_*DSP*_ = 0.5 s. For these optimizations, the convex cost function's objective was to minimize the synergy activation for the full duration and the final position error from *t* = *t*_*step*_ to *t* = *t*_*DSP*_. The cost function is defined as

(9) $$\begin{array}{l} \underset{c,M_{w}}{\text{min}}\Pi =\overset{t_{f}}{\underset{t_{0}}{\int }}c{\left(t\right)}^{T}R_{2}c\left(t\right)dt+\overset{t_{f}}{\underset{t_{step}}{\int }}E_{2}{\left(t\right)}^{T}Q_{2}E_{2}\left(t\right)dt\\ +\Pi_{extra} \end{array}$$

$$\begin{array}{l} \text{subject to}:c\in \left[c_{l},c_{u}\right] \end{array}$$

where final position error is defined as *E* = *q*_*f*_ − *q*, *q*_*f*_ is the final joint positions for a complete step, $R_{2}\in {\mathbb{R}}^{2\times 2}$ is the positive-definite weight matrix for the synergy activation, $Q_{2}\in {\mathbb{R}}^{4\times 4}$ is the positive-definite weight matrix for the the joint angle errors, and the lower and upper bound on the synergy activations are defined as *c*_*l*_ and $c_{u}\in {\mathbb{R}}^{2}$. In the cost function *t*_0_ is the time in which the step begins and *t*_*f*_ is the final time for the step and is defined as *t*_*f*_ = *t*_*step*_ + *t*_*DSP*_. The last variable in the cost function, Π_*extra*_ is an additional cost that is activated when certain undesirable events occur in the solution, e.g., the foot drags on the ground or the swing leg overshoots.

**Figure 3:**
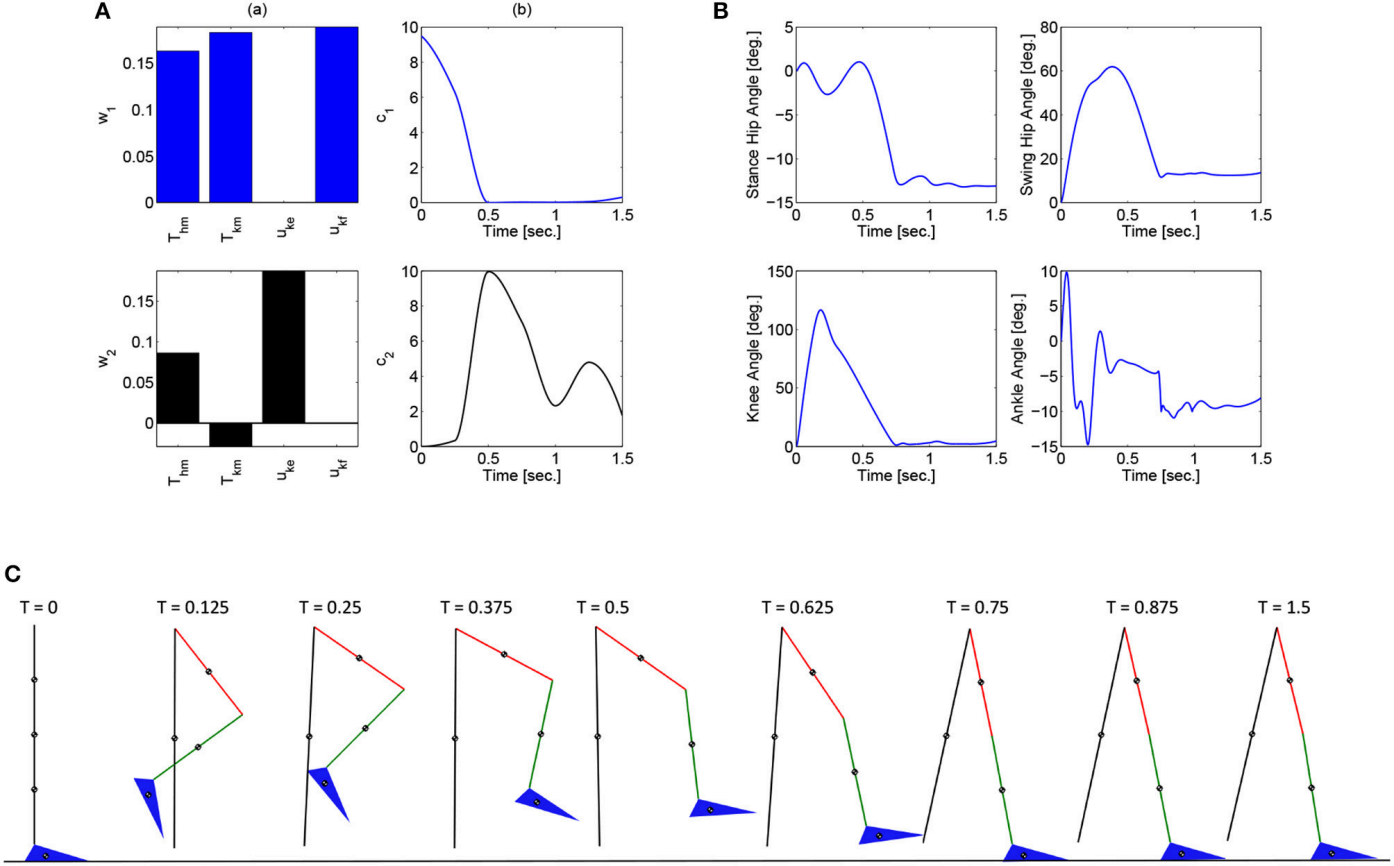
**(A)** The dynamic postural synergies (a) and their activation to produce a half step (b), **(B)** the joint trajectories they produce, **(C)** the gait sequence for the half step.

**Figure 4:**
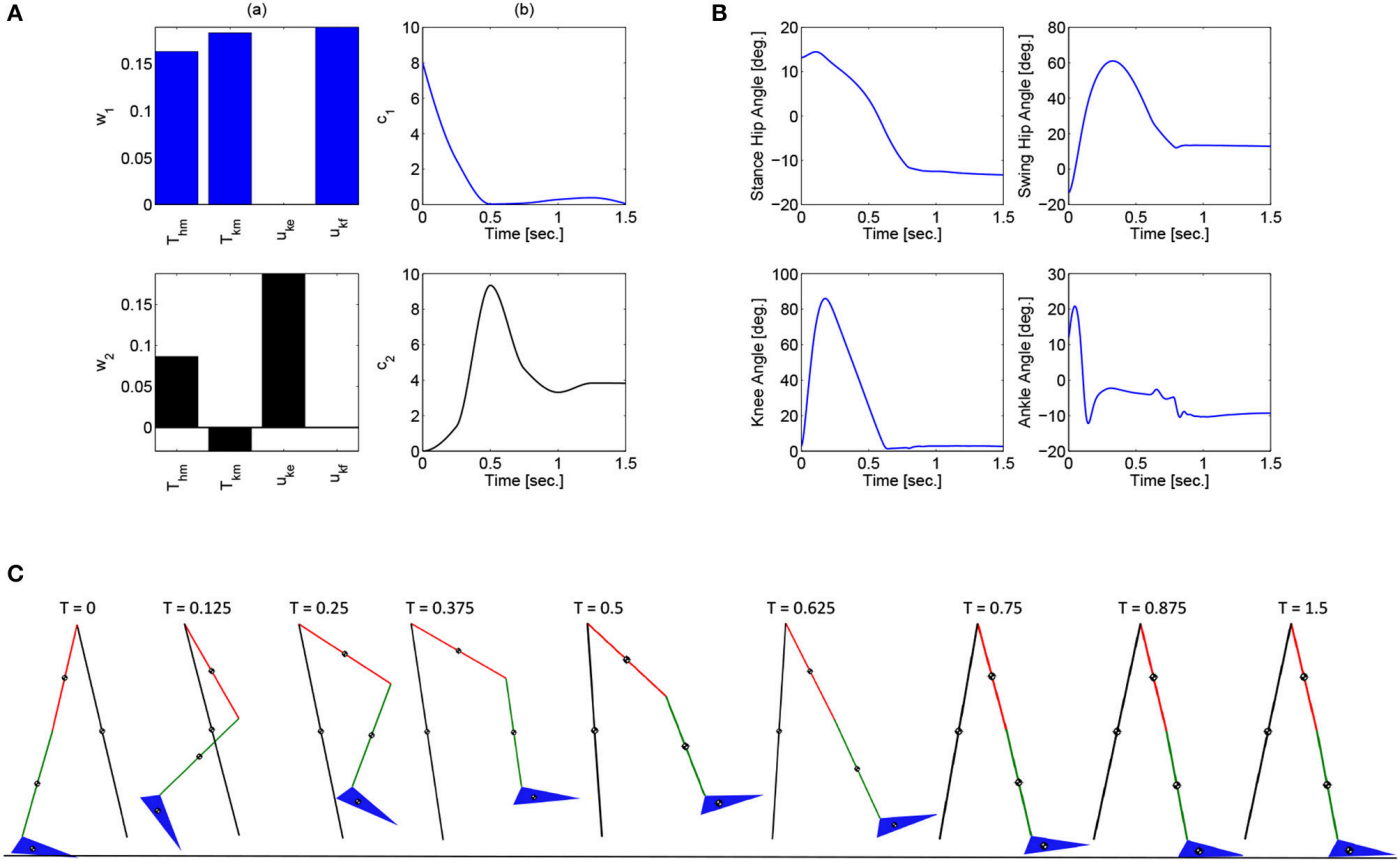
**(A)** The dynamic postural synergies (a) and their activation to produce a full step (b), **(B)** the joint trajectories they produce, **(C)** the gait sequence for the full step.

These optimizations were performed in Matlab using a genetic algorithm particle swarm optimization (GAPSO) method to minimize the cost function. The dynamic postural synergies, their activations computed through the optimizations, the joint trajectories they produce, and the gait sequence for the half step and full step can be seen in Figures 3, 4, respectively. From the gait sequences, it can be observed that the optimizations computed the synergy activations to complete the step, whether half or full, and maintained contact with the ground throughout the DSP while interacting with the ground reaction model. In addition, it can be seen that the dynamic postural synergies are activated in sequence as intended, i.e., for the first 0.5 s. primarily the first synergy is activated and then for the remainder of time primarily the second synergy is activated. Even though the model completes the step by around 1 s. the second synergy is still activated for the remainder of the time; this is to keep the knee from buckling since both legs are supporting the body during this phase.

Note that for the full step results, as the swing leg leaves the ground, the stance leg is tilted posteriorly which is not typical for normal gait. This is because this system does not currently include actuation at the ankle joints to produce push off. During normal gait the first part of the gait sequence is push off, as a result of the plantar-flexion of the ankle, to propel the body forward. The differences between gait with and without push off can be seen when comparing these results to the walking simulation results in Alibeji et al. (2015b) where ankle actuation is present. If the push off phase is to be included in this system, it would have its own dynamic postural synergy.

### 2.3. Control development and stability analysis

#### 2.3.1. Control objective

The control objective is to track a continuously differentiable desired trajectory $q_{d}\in {\mathbb{R}}^{4}$. The tracking error, *e* ∈ ℝ^4^, is defined as

(10) $$\begin{array}{l} e≜q_{d}-q. \end{array}$$

To facilitate the control design and stability analysis, the auxiliary error signals $e_{1}\left(t\right),r\left(t\right)\in {\mathbb{R}}^{4}$ are defined as

(11) $$\begin{array}{l} e_{1}≜{ė}_{0}+\alpha_{0}e_{0}, \end{array}$$

(12) $$\begin{array}{l} r≜{ė}_{1}+\alpha_{1}e_{1}, \end{array}$$

where $\alpha_{0},\alpha_{1}\in {\mathbb{R}}^{+}$ are control gains and $e_{0}\left(t\right)\in {\mathbb{R}}^{4}$ is an auxiliary signal defined as Downey et al. (2015)

(13) $$\begin{array}{l} e_{0}≜\int_{t_{0}}^{t}e\left(s\right)ds, \end{array}$$

in order to incorporate integral control. To simplify the derivations, the following notations are used: (1) the time dependence of a function is dropped [e.g., *e*(*t*) → *e*] and (2) a signal delayed by τ is notated as a subscript [e.g., *u*(*t* − τ) → *u*_τ_]. In addition, to facilitate the control development and stability analysis, the following assumptions were made.

**Assumption 1:** Only motion in the sagittal plane is considered.

**Assumption 2:** The unmodeled effects or disturbances, τ_*d*_, are bounded as |τ_*d*_| ≤ ϵ_1_ where $\epsilon_{1}\in {\mathbb{R}}^{+}$ is a constant.

**Assumption 3:** The dynamic postural synergies, *W*, are bounded constants and their activation, *c*_*d*_, are bounded vectors.

**Assumption 4:** The desired trajectory, $q_{d}\in {\mathbb{R}}^{n}$, and its derivatives, ${\dot{q}}_{d},{\ddot{q}}_{d}\in {\mathbb{R}}^{n}$, are bounded.

#### 2.3.2. Closed-loop error system

The open-loop error is derived by multiplying the time derivative of (12) with *M*(*q*) and substituting the dynamics in (1) and (2) to obtain

(14) $$\begin{array}{l} Mṙ=M\ddot{q_{d}}+C\dot{q}+G+f+d-b\phi \mu +M\alpha_{0}{ë}_{0}+M\alpha_{1}{ė}_{1}. \end{array}$$

where *d* is the lumped disturbances and is defined as *d* = Γ_*d*_ + Γ_*ext*_. This expression can be written in the form

(15) $$\begin{array}{l} Mṙ=-Cr+Ñ+N_{d}+d-b\phi \mu -e_{1}-b_{d}\phi e_{I}, \end{array}$$

where $b_{d}=b\left(q_{d},{\dot{q}}_{d}\right)$, $e_{I}\in {\mathbb{R}}^{4}$, is defined as $e_{I_{i_{j}}}≜\int_{t-\tau_{i_{j}}}^{t}u_{i_{j}}\left(\theta \right)d\theta$ for each actuator and Ñ ∈ ℝ^4^, is defined as Ñ ≜ *N* − *N*_*d*_. The auxiliary signals $N\left(q,\dot{q},e,ė,e_{I},t\right)$ and *N*_*d*_(*t*) are defined as

$$\begin{array}{c} N≜M\ddot{q_{d}}+C\left({\dot{q}}_{d}+\left(\alpha_{0}+\alpha_{1}\right)e_{1}-\alpha_{0}^{2}e_{0}\right)+G+f\\ +M\alpha_{0}\left(r-\left(\alpha_{1}+\alpha_{0}\right)e_{1}+\alpha_{0}^{2}e_{0}\right)\\ +M\alpha_{1}\left(r-\alpha_{1}e_{1}\right)+e_{1}+b_{d}\phi e_{I}, \end{array}$$

$$N_{d}≜M\left(q_{d}\right){\ddot{q}}_{d}+C\left(q_{d},\dot{q_{d}}\right)\dot{q_{d}}+G\left(q_{d}\right)+f\left(q_{d},\dot{q_{d}}\right).$$

The term Ñ in (15) can be upper bounded by using the Mean Value Theorem as

(16) $$\begin{array}{l} \left\|Ñ\right\|\leq \rho_{1}\left(\left\|z\right\|\right)\left\|z\right\|, \end{array}$$

where ρ_1_(||*z*||) ∈ ℝ is a positive monotonic bounded function and *z* ∈ ℝ^16^ is defined as

$$z={\left[\begin{array}{cccc} e_{0}^{T} & e_{1}^{T} & r^{T} & e_{I}^{T} \end{array}\right]}^{T}.$$

Note that the auxiliary signal *N*_*d*_ is equal to the left hand side of the desired muscle dynamics in (7). Therefore, (15) can be rewritten as

(17) $$\begin{array}{l} Mṙ=-Cr+Ñ+D+b_{d}\mu_{d}-b\phi \mu -e_{1}-b_{d}\phi e_{I}, \end{array}$$

where $D=d-\Gamma_{ext}^{*}$. After adding and subtracting the terms $b_{d}\hat{\phi }\bar{\mu },$ $b_{d}\phi \bar{\mu },$ $b_{d}\phi \hat{\mu },$ *b*_*d*_ϕμ, and *b*_*d*_ϕμ_*f*_ where $\hat{\mu }\in {\mathbb{R}}^{4}$ and $\hat{\phi }\in {\mathbb{R}}^{4\times 4}$ are estimates of the activation state and the fatigue state, $\bar{\mu }\in {\mathbb{R}}^{4}$ is the desired activation to be later defined, and $\mu_{f}\in {\mathbb{R}}^{4}$ is a filtered desired activation, and rearranging the terms, (17) becomes

(18) $$\begin{array}{l} Mṙ=-Cr+b_{d}\phi S+b_{d}\phi y+Ñ+D+b_{d}\mu_{d}+\tilde{b}\phi \mu \\ +b_{d}\phi \tilde{\mu }+b_{d}\tilde{\phi }\bar{\mu }-b_{d}\hat{\phi }\bar{\mu }-e_{1}, \end{array}$$

where $\tilde{b}\in {\mathbb{R}}^{4\times 4}$ is defined as $\tilde{b}≜b_{d}-b$, $\tilde{\phi }\in {\mathbb{R}}^{4\times 4}$ is defined as $\tilde{\phi }≜\hat{\phi }-\phi$, and $\tilde{\mu }\in {\mathbb{R}}^{4}$ is defined as $\tilde{\mu }≜\hat{\mu }-\mu$.

The estimates of the activation and fatigue states in (4) and (5) are generated through the following dynamics

(19) $$\begin{array}{l} {\dot{\hat{\mu }}}_{i_{j}}=-{ŵ}_{i_{j}}{\hat{\mu }}_{i_{j}}+{ŵ}_{i_{j}}u_{i_{j}}\left(t-\tau_{i_{j}}\right), \end{array}$$

(20) $$\begin{array}{l} {\dot{\hat{\phi }}}_{k_{j}}=\frac{1}{{\hat{T}}_{f_{k_{j}}}}\left({\hat{\phi }}_{\min_{k_{j}}}-{\hat{\phi }}_{k_{j}}\right){\hat{\mu }}_{k_{j}}+\frac{1}{{\hat{T}}_{r_{k_{j}}}}\left(1-{\hat{\phi }}_{k_{j}}\right)\left(1-{\hat{\mu }}_{k_{j}}\right), \end{array}$$

where ${ŵ}_{i_{j}},{\hat{T}}_{f_{k_{j}}},{\hat{T}}_{r_{k_{j}}},\text{and}{\hat{\phi }}_{{\text{min}}_{k_{j}}}$ are bounded estimates of the real parameters that can be determined through system identification experiments (Kirsch, 2016; Alibeji et al., 2017). Note that these estimators are governed by first-order differential equations, thus the estimates are bounded as $\hat{\mu }\in \left[u_{\text{min}},u_{\text{max}}\right]$ and $\hat{\phi }\in \left[{\hat{\phi }}_{\text{min}},1\right]$.

In (18), the surface error, *S* ∈ ℝ^4^, is defined as

(21) $$\begin{array}{l} S≜\mu_{f}-\hat{\mu }-e_{I}. \end{array}$$

The delay compensation term, *e*_*I*_, is added to the surface error, *S*, to deal with the input delay in the actuator dynamics. The boundary layer error, *y* ∈ ℝ^4^, for μ is defined as

(22) $$\begin{array}{l} y≜\bar{\mu }-\mu_{f}. \end{array}$$

The filtered desired activation μ_*f*_ is obtained by passing $\bar{\mu }$ through a low-pass filter such as

(23) $$\begin{array}{l} \zeta_{f}{\dot{\mu }}_{f}+\mu_{f}=\bar{\mu };\mu_{f}\left(0\right)=\bar{\mu }\left(0\right), \end{array}$$

where $\zeta_{f}\in {\mathbb{R}}^{+}$ is the low-pass filter time constant.

To felicitate the control design the desired activation, $\bar{\mu }$, is defined as

(24) $$\begin{array}{l} \bar{\mu }={\hat{\phi }}^{-1}\left[\zeta_{sf}Wĉ+kr\right], \end{array}$$

where ĉ ∈ ℝ^2^ is the estimate of *c*_*d*_, $\zeta_{sf}\in {\mathbb{R}}^{4\times 4}$ is a control gain matrix and *k* ∈ ℝ^4×4^ is the feedback gain matrix that is chosen to only influence the electric motors.

In $\bar{\mu }$, the feedforward component, ζ_*sf*_*Wĉ*, and the feedback component, *kr*, are scaled by the inverse of the fatigue estimate. This feature is included in the controller so that as a muscle fatigues, the stimulation input to that muscle increases gradually to counteract the effects of the fatigue. The estimate of the synergy activation updates according to the following update law with the projection algorithm (Dixon et al., 2003).

(25) $$\begin{array}{l} \dot{ĉ}=\text{proj}\left({ċ}_{d}+FW^{T}\zeta_{sf}^{T}b_{d}^{T}r\right), \end{array}$$

where *F* ∈ ℝ^2×2^ is a symmetric positive definite gain matrix. After using (24), (18) becomes

(26) $$\begin{array}{l} Mṙ=-Cr+b_{d}\phi S+b_{d}\phi y+Ñ+D+b_{d}\zeta_{sf}W\tilde{c}\\ +b_{d}\left(I-\zeta_{sf}\right)Wc_{d}+\tilde{b}\phi \mu +b_{d}\phi \tilde{\mu }\\ +b_{d}\tilde{\phi }{\hat{\phi }}^{-1}\zeta_{sf}Wĉ+b_{d}\tilde{\phi }{\hat{\phi }}^{-1}kr-b_{d}kr-e_{1}, \end{array}$$

where *I* is the identity matrix and $\tilde{c}\in {\mathbb{R}}^{2}$ is defined as

$$\tilde{c}=c_{d}-ĉ.$$

Using the Mean Value Theorem, Assumption 4, and the property of projection algorithm the following terms can be bounded as

(27) $$\begin{array}{l} \left\|\tilde{b}\phi \mu \right\|\leq \rho_{2}\left(\left\|z\right\|\right)\left\|z\right\|,\left\|b_{d}\right\|\leq \zeta ,\left\|D\right\|\leq \epsilon_{1} \end{array}$$

$$\begin{array}{l} \left\|b_{d}\left(I-\zeta_{sf}\right)Wc_{d}\right\|\leq \epsilon_{2},\left\|b_{d}\tilde{\phi }{\hat{\phi }}^{-1}\zeta_{sf}Wĉ\right\|\leq \epsilon_{3} \end{array}$$

where ρ_2_(||*z*||) ∈ ℝ is a positive monotonically increasing bounded function and $\epsilon_{1},\epsilon_{2},\epsilon_{3},\zeta \in {\mathbb{R}}^{+}$ are constants.

The surface error dynamics are derived by taking the time derivative of (21) and using (19), resulting in

(28) $$\begin{array}{l} Ṡ={\dot{\mu }}_{f}+ŵ\hat{\mu }-ŵu_{\tau }-\left(u-u_{\tau }\right). \end{array}$$

Based on the subsequent stability analysis, the normalized input *u* is designed as

(29) $$\begin{array}{l} u=\beta S+{\dot{\mu }}_{f}, \end{array}$$

where β ∈ ℝ^+^ is a control gain.

Therefore, the closed-loop surface error dynamics can be written as

(30) $$\begin{array}{l} Ṡ=-\beta S+ŵ\hat{\mu }+\left(1-ŵ\right)u_{\tau }. \end{array}$$

**Figure 5:**
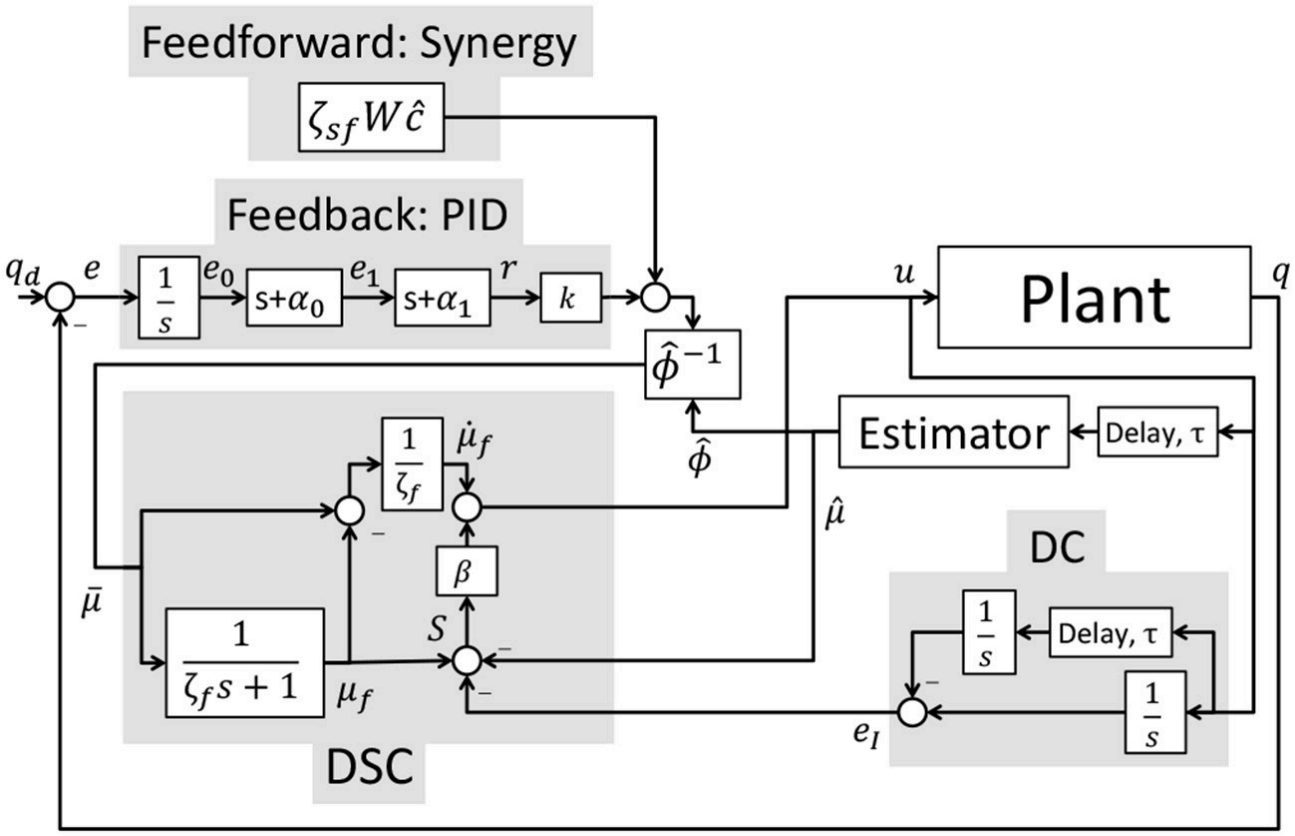
The control schematic for the implementation of the overall controller.

The boundary layer error dynamics are found by taking the time derivative of (22) and using (23), which results in

(31) $$\begin{array}{l} ẏ=\eta -\frac{y}{\zeta_{f}}, \end{array}$$

where η(*e, r, S, y, t*) is a continuous nonlinear function defined as $\eta =\frac{d}{dt}\left[\bar{\mu }\right].$ Based on the definition of *u* in (29), the control law *v* is designed as

(32) $$\begin{array}{l} v=\left[\beta S+\frac{{\hat{\phi }}^{-1}\left[\zeta_{sf}Wĉ+kr\right]-\mu_{f}}{\zeta_{f}}-u_{min}\right]\frac{△v}{△u}+v_{\min}, \end{array}$$

where △*v* = *v*_max_ − *v*_min_ and △*u* = *u*_max_ − *u*_min_. The desired feedback activation, *kr*, defined in (24) can be expressed in standard PID form as $K_{P}e+K_{D}ė+K_{I}\int_{0}^{t}e\left(\theta \right)d\theta$ where $K_{P},K_{D},K_{I}\in {\mathbb{R}}^{+}$ are the proportional, derivative, and integral control gains and are defined as *K*_*P*_ = *k*(α_0_ + α_1_), *K*_*D*_ = *k*, and *K*_*I*_ = *kα*_0_α_1_. The control schematic for the implementation of the overall controller is represented in Figure 5.

### 2.4. Finite state machine

**Figure 6:**
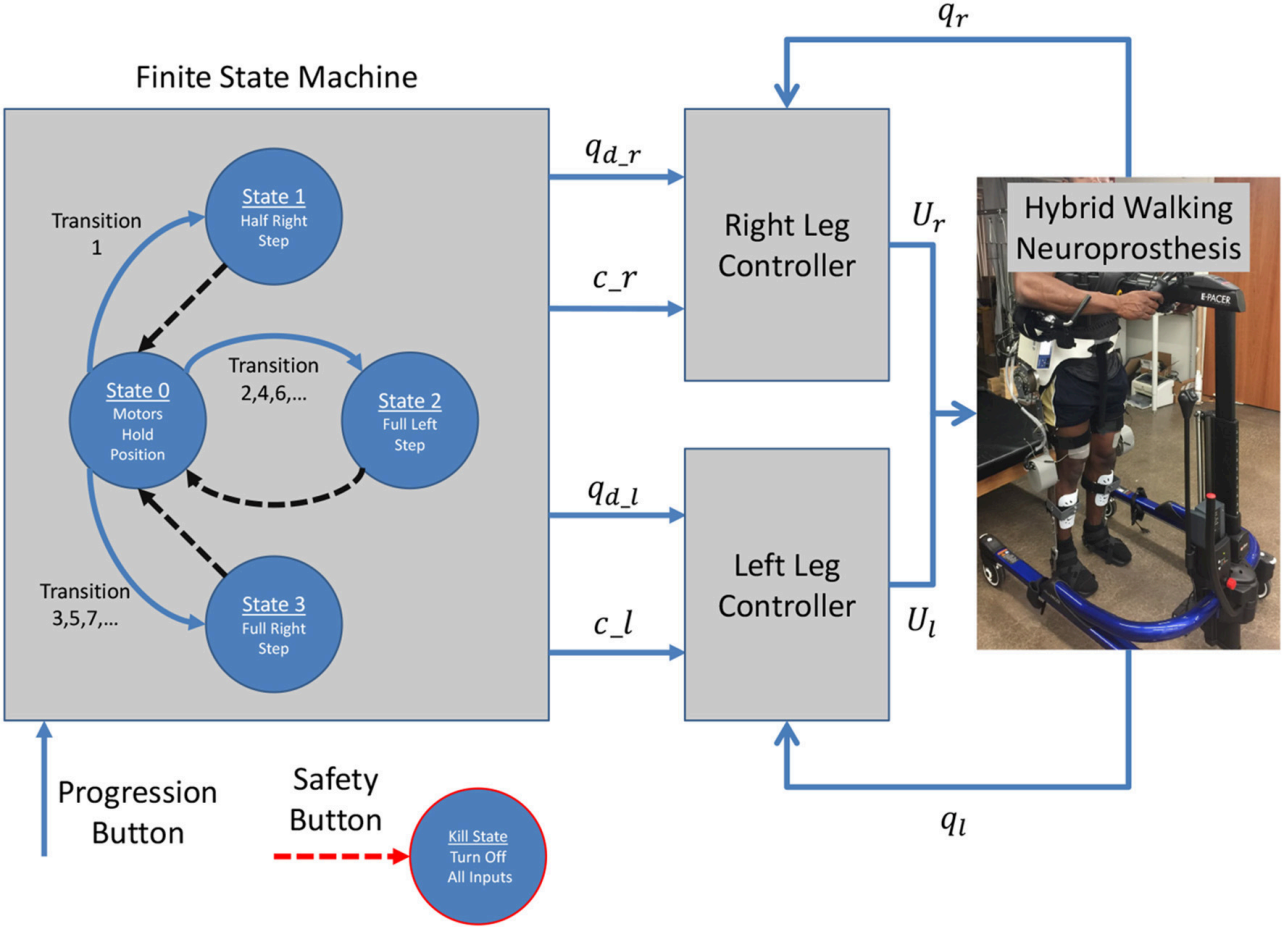
The Finite State Machine determines the desired trajectories and synergy activations based on what state is activated; either half right step, full left step, or full right step. Then two controllers are used, one for each leg, which work in tandem to produce gait.

The hybrid neuroprosthesis used for experimental demonstration uses 4 electric motors; one on each hip joint and knee joint, and 4 stimulation channels; the quadriceps and hamstrings of each leg. The hybrid neuroprosthesis is controlled using two of the adaptive synergy-based PID-DSC controller with delay compensation working in tandem to produce gait, one for each leg. The Finite State Machine, shown in Figure 6, is used to determine which trajectories and synergy activations of the gait sequence are used; i.e., either half right step (State 1), full left step (State 2), or full right step (State 3). In between the active states; State 1–3, the standby state (State 0) is activated by default, in which the motors at the joints hold their positions and the synergy activations are set to zero. When a leg is activated in a state, it becomes the swing leg and its counterpart becomes the stance leg. When a leg becomes the stance leg the controller only uses feedback to track the stance hip trajectory and hold the position of the knee joint. The progression of the FSM is determined by the progression button, in which the first time it is pressed State 1 is activated, then each time it is pressed after that the even transitions activate State 2 and the odd transitions activate State 3. In addition to the progression button, there is a safety button which turns off all inputs when pressed.

### 2.5. Experimental demonstration

**Figure 7:**
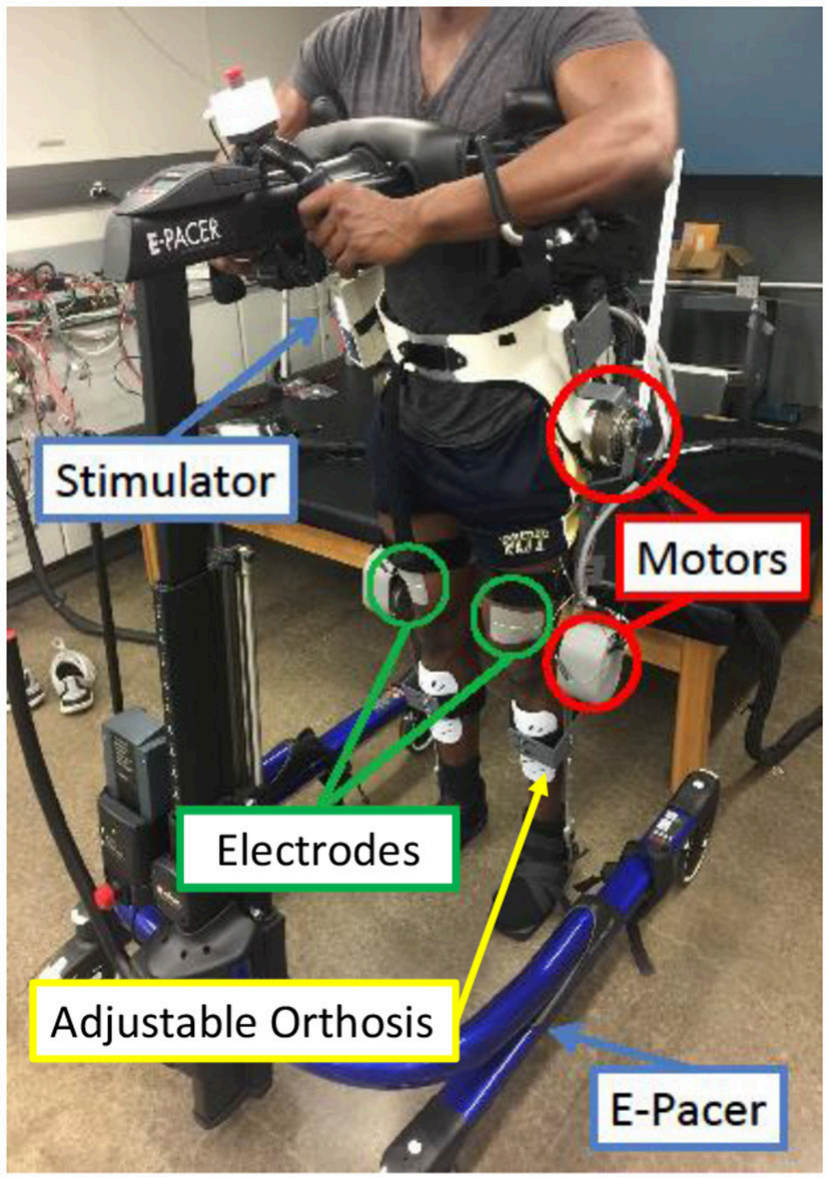
The walking hybrid neuroprosthesis and the gait support device used in the experimental demonstration of the synergy-based control system. This system uses an electric motor at the hip and knee joints of each leg and FES of the hamstrings and quadriceps muscle group of each leg.

The hybrid neuroprosthesis testbed, shown in Figure 7, can be broken down into four primary components: an adjustable orthosis, electric motors, a stimulation unit, and an assistive support device. The orthosis is designed to be adjustable to comfortably fit a wide variety of body types while maintaining the alignment of the joints between the orthosis and subject. Custom motor mount brackets were fabricated to attach the electric motors at the joints of the orthosis. The electric motors (Harmonic Drive LLC, MA, USA) at the hip joints can generate a maximum torque of 50 Nm. The knee electric motor were EC90 brushless motors (Maxon Motor, Switzerland) combined with a Harmonic Gear CSD-25-100-2UH (Harmonic Drive LLC, MA, USA). The knee motor can generate a maximum torque of 56 Nm. A RehaStim 8-channel stimulator (Hasomed Inc., DE) was used to generate the current modulated biphasic pulse trains used to elicit muscle contractions. A set of transcutaneous electrodes was placed on the quadriceps and hamstring muscle groups. The current modulated pulse train with a frequency of 35 Hz and a 400 μs pulse width is typically used for all experiments. An assistive support device, called an E-Pacter (Rifton, USA), is used for the experiments to help the subjects maintain their balance and propel themselves forward. An xPC target (SpeedGoat, CH) was used to interface with the different sensors and motor drivers and implement the controller in real-time at 1 kHz. The control algorithms were coded in Simulink (MathWorks Inc, USA) and used Simulink's (MathWorks Inc, USA) real-time toolbox software running on a Windows machine (Intel Xeon 3.10 GHz processor). The hybrid neuroprosthesis is controlled using a button to control the progression of gait and an emergency stop button to stop all the inputs.

The overall control system was experimentally demonstrated on an able-bodied subject (male; 27 years old, height: 1.80 m, weight: 90 kg) and a person with an incomplete SCI (male; 41 years old, height 1.70 m, weight 70 kg, injury: T10 AIS A). For these experiments it is assumed that the behavior of the right and left leg are similar, therefore, both States 2 and 3 use the same synergies and activations computed in the previous sections. The optimizations to compute the synergies, their activations, and the trajectories they produce were performed using the subject's height and weight, but the model used the muscle parameters reported in Popović et al. (1999) for an able-bodied subject and person with SCI, respectively. If this system is to be implemented on a subject with a condition in which a injury/disorder in which one of his or her leg's response is much different than his or her other leg such as in hemiplegia due to a stroke, it would probably be more beneficial to use multiple subject-specific models, one for each leg.

Prior to any experimentation, an approval from the Institutional Review Board at the University of Pittsburgh was obtained. The consent procedure for human participants was written and informed. During the experiments, the subject was instructed to relax and refrain from voluntarily interfering with the hybrid exoskeleton. The estimates of the EMD, activation time constants, and fatigue/recovery rates were estimated in system identification experiments in a leg extension machine and assumed to be the same for both legs. During the experiments, the subjects used a gait assistive device called the E-Pacer (Rifton, USA) to help support and propel themselves forward. The progression and safety buttons were operated by a separate user and were used to control the FSM. The experiments were run for 6 steps, including the half right step. In order to compare the difference in power consumption between a powered exoskeleton, just motors, and a hybrid neuroprosthesis, motors and FES, the testbed was tested with two different control systems. For the first control system for the hybrid neuroprosthesis configuration, the adaptive synergy-based PID-DSC controller was used to govern the input to the FES and motors. For the second control system for the powered exoskeleton configuration, a Robust Integral of the Sign of the Error (RISE) (Xian et al., 2004) controller was used to govern the input to the motors. This controller was used for this case because it contains a unique integral signum term which can accommodate for sufficiently smooth bounded disturbances like the friction in the harmonic drive motors used in this testbed.

## 3. Results

**Figure 8:**
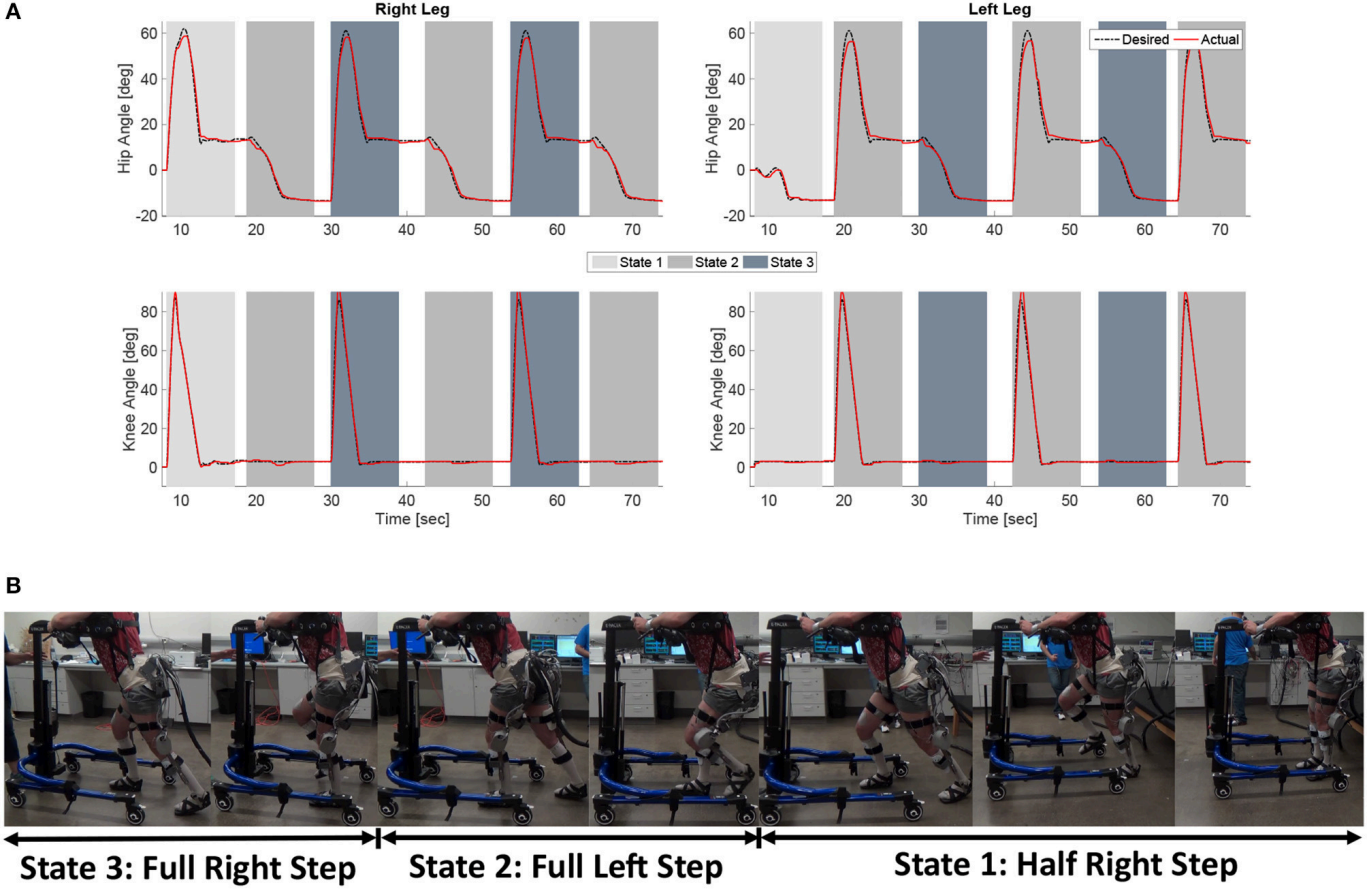
**(A)** The desired and actual joint angles of the right and left hip and knee joints resulting from using the developed synergy-based DSC/DC control system in conjunction with the FSM on a subject with an incomplete SCI. The shaded regions indicate which state of the FSM is active at that time. **(B)** A sequence of photos illustrating the gait produce during the experiments. The depicted individual provided written and informed consent for the publication of this image.

**Figure 9:**
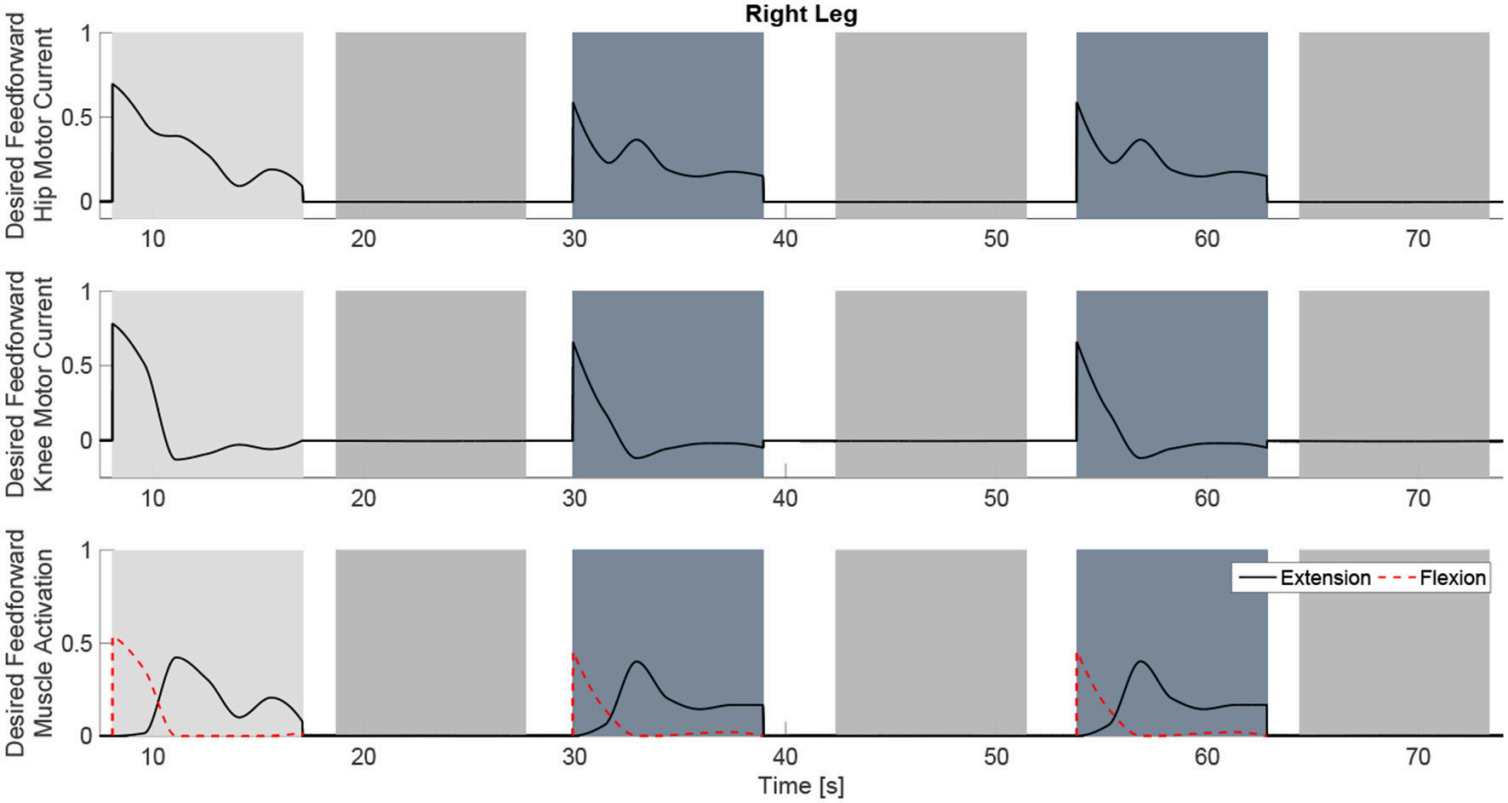
The desired feedforward component of $\bar{\mu }$ for all of the system inputs. This component is generated from the dynamic postural synergies and their activation after adaptation and with the scaling up from the fatigue estimate and the scaling factor control gain.

**Figure 10:**
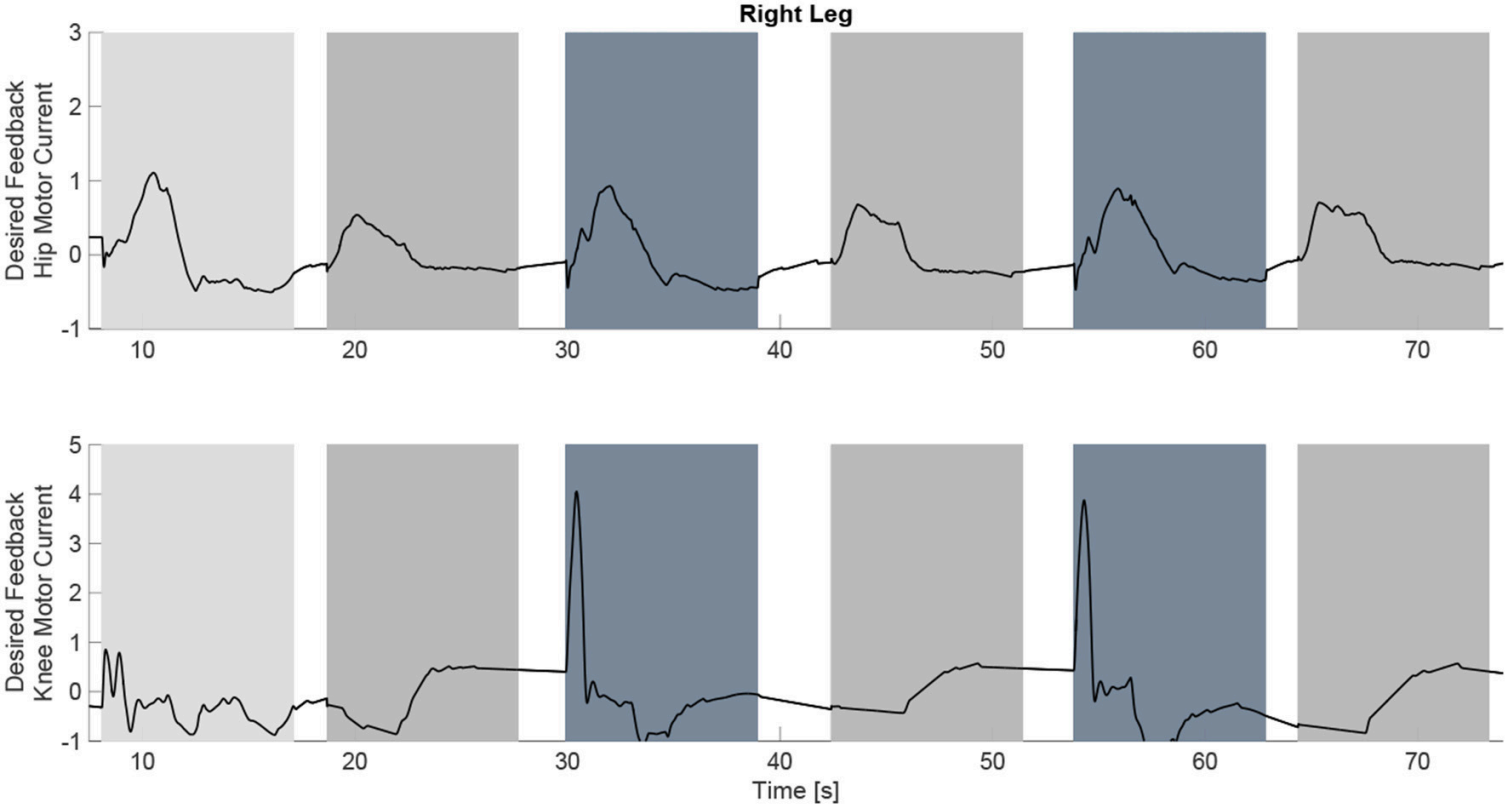
The desired feedback component of $\bar{\mu }$ which is only applied to the four motors at the hip and knee joints of each leg. It can be observed that they majority of the effort is occurring during the swing phase of each leg.

**Figure 11:**
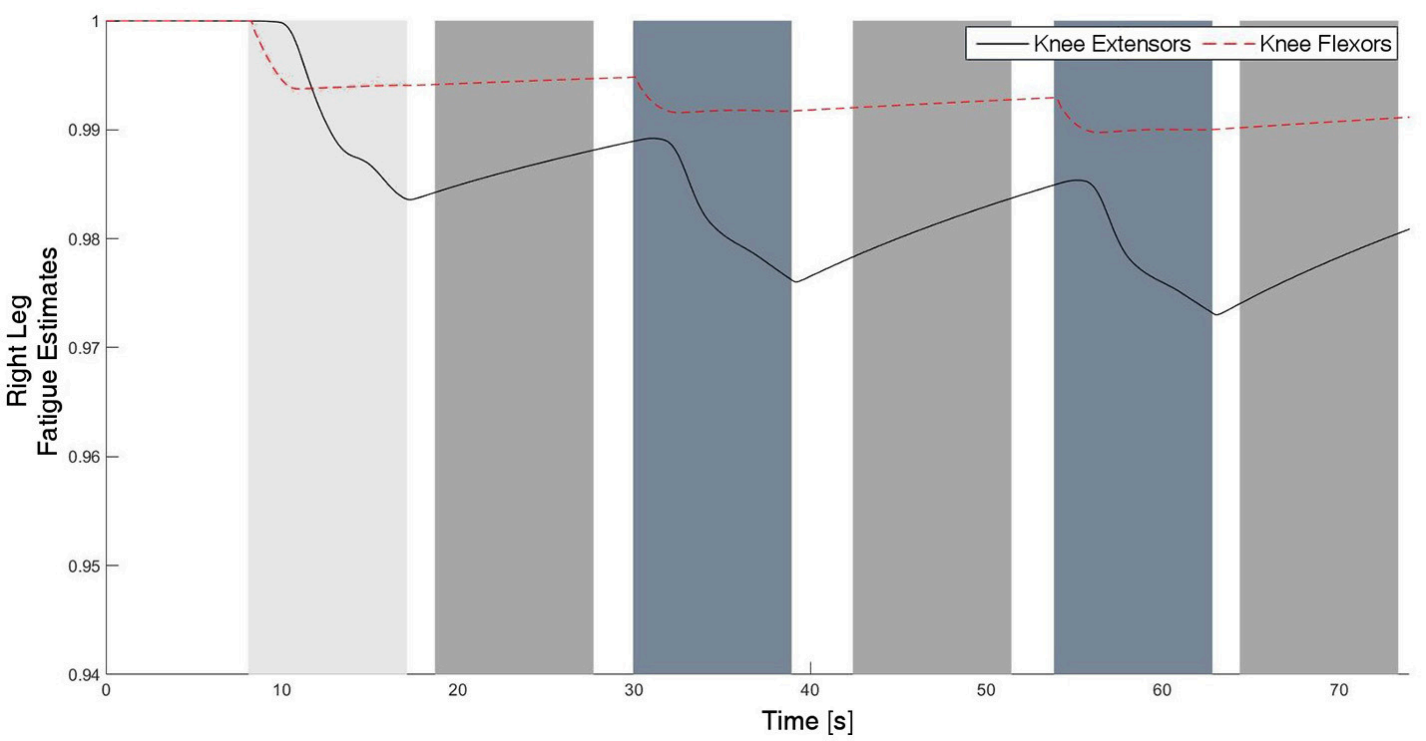
The fatigue estimates for the knee flexors and extensors of the right leg. The fatigue estimate ranges from 1 to ϕ_*min*_, which corresponds to no fatigue to fully fatigued, respectively. It can be observed that the fatigue occurs during the swing phase, and the muscles recover during the stance phase since there is no stimulation.

**Figure 12:**
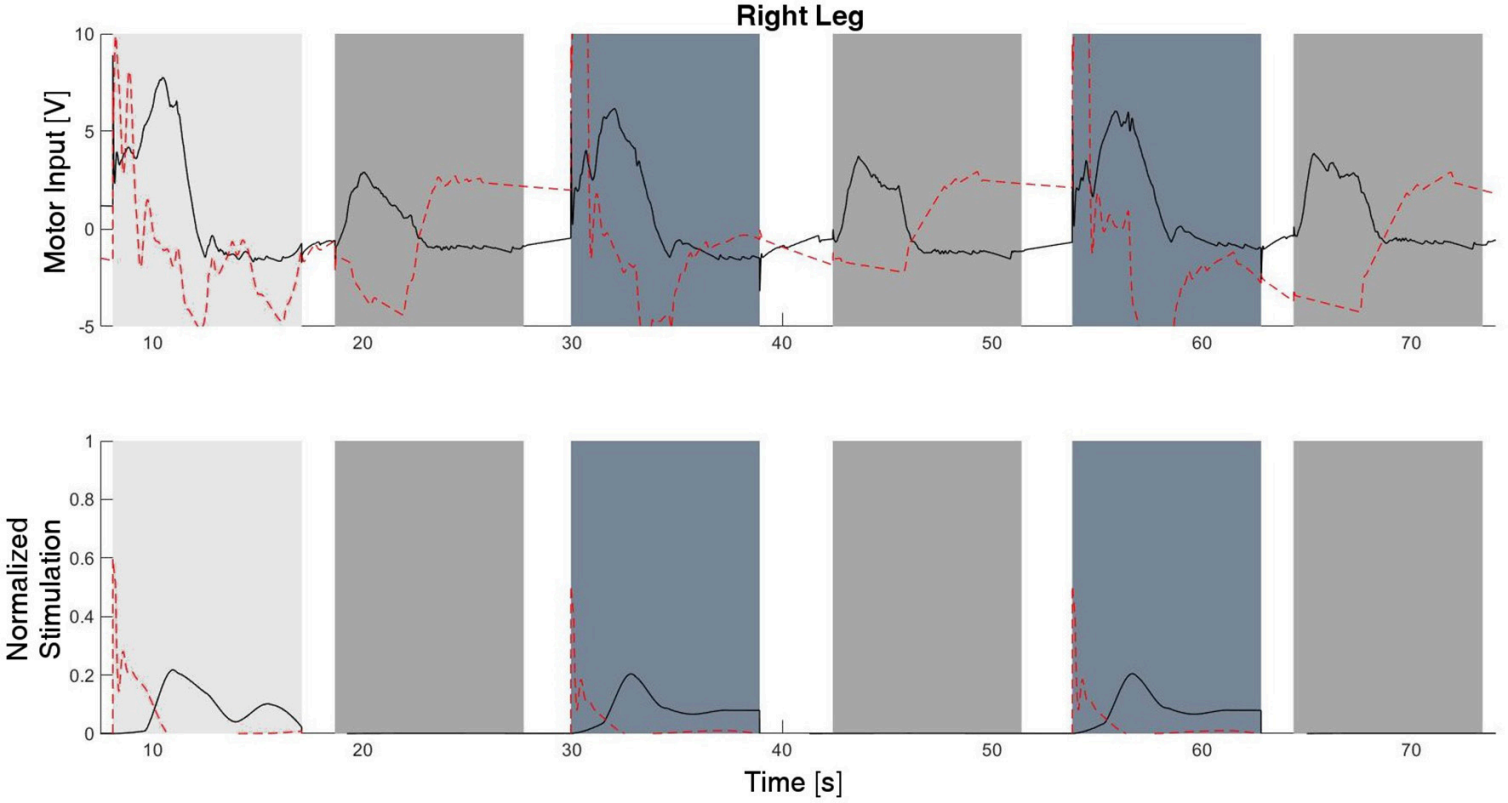
The inputs to all of the system inputs, including feedback and feedforward, for this experimental trial. Note that there is no stimulation occurring during the stance phase of each leg.

The experimental results from the subject with the incomplete SCI can be seen in Figures 8–12. The tracking performance for the both right and left hip and knee joints can be seen in Figure 8A. Figure 8B shows a sequence of frames from the video footage illustrating the gait produced using the control system^1^. The root mean squared errors (RMSE) and root mean squared voltages (RMSV) for the hip and knee joints for the right leg are presented in Table 1. From the results it can be seen that not only did the synergy-based controller result in better tracking performance, but it did so while consuming less energy compared to the RISE controller. In addition, the hybrid neuroprosthesis testbed, when using the synergy-based controller, also includes theraputic health benefits due to the use of FES. The desired feedforward component, ${\hat{\phi }}^{-1}\zeta_{sf}Wĉ$, and desired feedback component, *kr*, in $\bar{\mu }$ can be seen in Figures 9, 10. The contribution of the inverse of the fatigue estimate scaling factor is not apparent in the experimental results as there is little change in the desired feedfoward activations, as seen in Figure 9. This is due to the small changes in the estimate of the fatigue, as seen in Figure 11. This is due to the fatigue parameters identified for the subject with an incomplete SCI. Since his injury level is incomplete, his muscles had not atrophied and resistant to fatigue. However, for the subjects with advanced muscle atrophy as a result of their complete SCI, muscle fatigue would occur more rapidly, hence this is still a practical feature in the controller. The actual input signals for all 8 inputs of the system can be seen in Figure 12. It can be observed, that when a leg takes the role of the stance leg, the synergy activation is zero which results in zero stimulation and zero desired feedforward motor activation. Hence, only feedback control of the motors is used to lock the knee joint of the stance leg. From the inputs, we can see that the timing of the stimulation is sensible as for each step the flexors is activated first to produce the withdrawal reflex and then the extensors to fully extend the knee.

**Table 1:** The root mean squared of the input voltage to the motors.

**Subject**	**Joint**	**Synergy-based Controller**		**RISE**	
		**RMSE [deg.]**	**RMS V [V]**	**RMSE [deg.]**	**RMSV [V]**
Incomplete SCI	Right Hip	1.35	2.25	1.68	2.49
	Right Knee	1.68	3.10	3.52	3.36
Able Bodied	Right Hip	1.56	3.22	2.70	3.39
	Right Knee	0.92	2.50	3.03	3.70

*Note that the Synergy-based controller uses less motor input which means less power consumption and results in better tracking performance*.

## 4. Discussion

As researchers, we often analyze biological systems to devise innovative solutions to real world applications. To overcome the challenge of actuator redundancy, we studied how scientists believed the human body solves its high degree of freedom and actuator redundancy problem to achieve fluid and coordinated movements such as gait. It is hypothesized that the human central nervous system (CNS) activates multiple muscle fibers in groups or patterns called muscle synergies, or motor primitives, to efficiently perform complex movements such as reaching, hand manipulations, or posture control (Sherrington, 1910; d‘Avella and Tresch, 2001; Ting, 2007; Vinjamuri, 2008; Vinjamuri et al., 2010). The benifit of synergies is their function of transforming a higher dimensional and complex systems into lower dimensional and simpler systems that are easier to control (Tresch and Jarc, 2009). In Neptune et al. (2009), muscle synergies for human locomotion were extracted and successfully applied to complex human walking models to reproduce realistic gait motions. For a more thorough literature review on synergies, readers are referred to these references (Vinjamuri, 2008; Tresch and Jarc, 2009).

In this research, a synergy-based control system is used to distribute the control effort to the multiple actuators of a walking hybrid neuroprosthesis. This approach is inspired from the human motor control concept of muscle synergies. In most studies, muscle synergies are proposed as a basis employed during human motor control and found by decomposing recorded EMG signals (collected from multiple muscles) to extract muscle synergies. Unlike these studies, in this paper dynamic postural synergies are designed, using dynamic optimizations, to be used as a basis for the control system for the walking hybrid neuroprosthesis. This synergy design approach, using optimizations to distribute the control effort among the available actuators, offers multiple advantages and convenience such as allowing for the incorporation of external inputs, i.e., electric motors and FES. Another benefit for this method of designing dynamic postural synergies is the ease of adding additional restrictions on the synergies, i.e., no co-activation or no negative stimulation. Based on the synergy principle, fewer control signals are used to control multiple actuators in a hybrid neuroprosthesis, therefore the use of synergies will not only solve the actuator redundancy problem similarly to how the body is hypothesized to do so, but it will do it in a more computationally efficient way. However, there are still other remaining challenges that could hamper the effectiveness of a closed-loop synergy-based control system if not addressed. These remaining challenges are EMD, actuator dynamics, and muscle fatigue. Therefore, Lyapunov-based control design approaches were used to derive this class of synergy-based controllers that are robust to EMD and compensate for activation dynamics and muscle fatigue. While the developed control system was capable of reproducing gait, the finite state machine can still be scaled-up to achieve motions other than gait such as sitting/standing and ascending/descending.

## 5. Conclusion

In this paper, the adaptive synergy-based DSC controller is developed and experimentally tested on an able-bodied subject and person with an incomplete SCI using a walking hybrid neuroprosthesis. This control system used dynamic postural synergies designed to reproduce the key dynamic posture; the withdrawal reflex and knee extension, which have been shown to be able to reproduce gait. Dynamic optimizations were then used to compute the optimal synergies' activation to produce a half step and full step. A finite state machine was developed to switch between the trajectories and synergy activations depending on three states; half right step, full right step, and full left step. The control system then used two of the synergy-based DSC controller, one for each leg, working in tandem to reproduce gait. The overall control system was able to recreate gait using the hybrid neuroprosthesis and the gait assistive device.

## Author contributions

NA designed the controller, developed dynamic postural synergies, performed experiments, and wrote the paper. VM performed optimizations, BD recruited subjects for the study and supervised and provided advise on conducting experiments with subjects with SCI, and NS designed and conceptualized the control design, study, experiments, and edited the manuscript.

### Conflict of interest statement

The authors declare that the research was conducted in the absence of any commercial or financial relationships that could be construed as a potential conflict of interest.

## Footnotes

^1^The video footage of testing of the dynamic postural synergy-based controller on a subject with an incomplete SCI can be seen in the Supplementary Files.
