# Supplementary material for: A Control Scheme That Uses Dynamic Postural Synergies to Coordinate a Hybrid Walking Neuroprosthesis: Theory and Experiments
Source: Front Neurosci. 2018 Apr 10;12:159. doi: 10.3389/fnins.2018.00159 (PMC5902565; doi:10.3389/fnins.2018.00159)
Supplement: Supplementary file 2 [file DataSheet1.pdf]

APPENDIX

Theorem 1. *The controller designed in* (24)*,* (25)*, and* (32)*, when $v_{min} \leq v \leq v_{max}$, ensures uniformly ultimately bounded tracking*

$$\|y(t)\| \leq \delta_0 \exp(-\delta_1 t) + \delta_2, \tag{33}$$

where $\delta_1, \delta_2, \delta_3 \in \mathbb{R}^+$ denote constants, provided that the following gain conditions are satisfied:

$$K_{min} > \frac{(\rho_1(\|z\|) + \rho_2(\|z\|))^2}{2\zeta},$$
$$\gamma_{min}\left\{b_d k - \Lambda\right\} > 0, \;\; \beta > \vartheta + \frac{\bar{\tau}\zeta_f}{\beta},$$
$$\alpha_0 > \frac{1}{2}, \;\; \alpha_1 > \frac{1}{2}$$

where $\gamma_{min}\{\cdot\}$ denotes the minimum eigenvalue of a square matrix and K_{min}, $\vartheta, \bar{\tau} \in \mathbb{R}^+$ are subsequently defined constants.

Proof: To prove stability, choose a continuously differentiable Lyapunov candidate $V(x,t) \in \mathbb{R}$, defined as

$$V = \frac{1}{2}e_0^T e_0 + \frac{1}{2}e_1^T e_1 + \frac{1}{2}r^T M r + \frac{1}{2}S^T S + \frac{1}{2}y^T y + \frac{1}{2}\tilde{c}^T F^{-1}\tilde{c} + P. \tag{34}$$

The Lyapunov candidate V can be lower and upper bounded as

$$\lambda_1 \|x\|^2 \leq V \leq \lambda_2 \|x\|^2 + \Upsilon, \tag{35}$$

where Υ, λ_1, $\lambda_2 \in \mathbb{R}^+$ are constants and $x = [\; e_0^T \;\; e_1^T \;\; r^T \;\; S^T \;\; y^T \;\; \sqrt{P} \;]^T$. The Lyapunov-Krasovskii functional $P \in \mathbb{R}^+$ is defined as

$$P = \frac{\zeta_f}{\beta^2}\int_{t-\bar{\tau}}^{t}\left(\int_{s}^{t} u(\theta)^{\mathrm{T}} u(\theta) d\theta\right) ds,$$

where $\bar{\tau} = \max(\tau_{i_j})$. Taking the time derivative of $V(x,t)$ and using (13), (11), (12), (26), (30), (31), the update law in (25), the skew-symmetry property [45] and canceling out the like terms results in

$$\begin{aligned}\dot{V} =& -\alpha_0 e_0^T e_0 - \alpha_1 e_1^T e_1 - r^T b_d k r - S^T \beta S - \frac{1}{\zeta_f} y^T y \\ &+ S^T\left(\hat{w}\hat{\mu} + (1-\hat{w})u_\tau\right) + e_0 e_1 + y^T \eta \\ &+ r^T\left(\tilde{N} + D + b_d(I - \zeta_{sf})W c_d + b_d \phi S\right) \\ &+ r^T\left(b_d \phi y + \tilde{b}\phi\mu\right) + r^T\left(b_d \tilde{\phi}\bar{\mu} + b_d \phi\tilde{\mu}\right) \\ &+ \frac{\zeta_f}{\beta^2}\bar{\tau}\|u\|^2 - \frac{\zeta_f}{\beta^2}\int_{t-\bar{\tau}}^{t}\|u(\theta)\|^2 d\theta.\end{aligned}$$

The previous equation can be bounded using (16), (27), the definitions of u, and Assumptions 2 and 3 to get

$$\begin{aligned}\dot{V} \leq& -\alpha_0 e_0^T e_0 - \alpha_1 e_1^T e_1 - r^T\left(b_d k - b_d \tilde{\phi}\hat{\phi}^{-1} k\right) r \\ &- S^T \beta S - \frac{1}{\zeta_f} y^T y + \|S\|\left\|\hat{w}\hat{\mu} + (1-\hat{w})u_\tau\right\| \\ &+ \|r\|\left[(\rho_1(\|z\|) + \rho_2(\|z\|))\|z\| + \epsilon_1 + \epsilon_2\right] \\ &+ \|e_0\|\|e_1\| + \|y\|\|\eta\| + \|b_d\phi\|\|r\|\|S\| \\ &+ \|b_d\phi\|\|r\|\|y\| + \|r\|\left\|b_d\tilde{\phi}\hat{\phi}^{-1}\zeta_{sf}W\hat{c}\right\| \\ &+ \|r\|\|b_d\phi\tilde{\mu}\| - \frac{\zeta_f}{\beta^2}\int_{t-\bar{\tau}}^{t}\|u(\theta)\|^2 d\theta \\ &+ \frac{\zeta_f\bar{\tau}}{\beta^2}\left(\beta^2\|S\|^2 + 2\beta\|S\|\|\dot{\mu}_f\| + \|\dot{\mu}_f\|^2\right).\end{aligned}$$

Using the fact that $u \in [u_{\text{min}}, u_{\text{max}}]$, $\hat{\mu} \in [u_{\text{min}}, u_{\text{max}}]$, and $\hat{\phi} \in [\hat{\phi}_{\text{min}}, 1]$, Young's inequality, and (27) to bound the following terms

$$\begin{aligned}\|e_0\|\|e_1\| \leq& \frac{1}{2}\|e_0\|^2 + \frac{1}{2}\|e_1\|^2, \\ \|b_d\phi\|\|r\|\|S\| \leq& \frac{\zeta}{2}\|r\|^2 + \frac{\zeta}{2}\|S\|^2, \\ \|b_d\phi\|\|r\|\|y\| \leq& \frac{\zeta}{2}\|r\|^2 + \frac{\zeta}{2}\|y\|^2, \\ \|y\|\|\eta\| \leq& \frac{1}{2\varepsilon}\|y\|^2\|\eta\|^2 + \frac{\varepsilon}{2}, \\ \|S\|\left\|\hat{w}\hat{\mu} + (1-\hat{w})u_\tau\right\| \leq& \Psi\|S\| \leq \frac{1}{2\varepsilon}\Psi^2\|S\|^2 + \frac{\varepsilon}{2}, \\ \|r\|\left\|b_d\tilde{\phi}\hat{\phi}^{-1}\zeta_{sf}W\hat{c}\right\| \leq& \frac{\epsilon_3^2}{2\varepsilon}\|r\|^2 + \frac{\varepsilon}{2} \\ \|b_d\phi\tilde{\mu}\|\|r\| \leq& \frac{\zeta^2}{2\varepsilon}\|r\|^2 + \frac{\varepsilon}{2} \\ \frac{2\bar{\tau}}{\beta}\|S\|\|y\| \leq& \frac{\bar{\tau}\zeta_f}{\beta}\|S\|^2 + \frac{\bar{\tau}}{\zeta_f\beta}\|y\|^2\end{aligned}$$

where $\varepsilon \in \mathbb{R}^+$ is an arbitrary constant, $\Psi = \hat{w} + (1+\hat{w})\,u_{max}$, and ζ is defined in (27). After rearranging the terms this expression becomes

$$\begin{aligned}\dot{V} \leq& -\left(\alpha_0 - \frac{1}{2}\right)e_0^T e_0 - \left(\alpha_1 - \frac{1}{2}\right)e_1^T e_1 \\ &- r^T\left(b_d k + \zeta - \mathit{\Lambda}\right) r - S^T\left(\beta - \vartheta - \frac{\tau\zeta_f}{\beta}\right) S \\ &- \left(\frac{\kappa}{\zeta_f} - \frac{\zeta}{2}\right) y^T y + \frac{1}{2\varepsilon}\|y\|^2\|\eta\|^2 \\ &+ \|r\|\left[(\rho_1(\|z\|) + \rho_2(\|z\|))\|z\| + \epsilon_1 + \epsilon_2\right] \\ &+ 2\varepsilon - \frac{\zeta_f}{\beta^2}\int_{t-\bar{\tau}}^{t}\|u(\theta)\|^2 d\theta.\end{aligned}$$

where $\mathit{\Lambda} = b_d\tilde{\phi}\hat{\phi}^{-1}k + \left(2\zeta + \frac{\zeta^2}{2\varepsilon} + \frac{\epsilon_3^2}{2\varepsilon}\right)I$, $\vartheta = \frac{\zeta}{2} + \frac{\Psi^2}{2\varepsilon} + \zeta_f\bar{\tau}$, and $\kappa = 1 - \bar{\tau}\beta^{-2} - \bar{\tau}\beta^{-1}$. After defining ζ_f such that

$$\frac{1}{\zeta_f} \geq \frac{1}{\kappa}\left[\frac{\zeta}{2} + \frac{\Omega^2}{2\varepsilon} + k_o\right], \tag{36}$$

where $k_o \in \mathbb{R}^+$ is a known constant and $\Omega > 0$ is the maximum of η in the defined compact set $\Xi =$

$\left\{h \in \mathbb{R}^{8n} | \, \|h\| < 2\sigma, h = [e,\ r,\ S,\ y]^T\right\}$ where $\sigma \in \mathbb{R}^+$ is a known constant, the previous equation becomes

$$\begin{aligned}\dot{V} \leq & -\left(\alpha_0 - \frac{1}{2}\right) e_0^T e_0 - \left(\alpha_1 - \frac{1}{2}\right) e_1^T e_1 \\ & - r^T \left(b_d k + \zeta - \mathit{\Lambda}\right) r - S^T \left(\beta - \vartheta - \frac{\bar{\tau}\zeta_f}{\beta}\right) S \\ & - k_o y^T y + \|r\| \left[\left(\rho_1(\|z\|) + \rho_2(\|z\|)\right) \|z\| + \epsilon_1 + \epsilon_2\right] \\ & - \left(1 - \frac{\|\eta\|^2}{\Omega^2}\right) \frac{\|y\|^2 \Omega^2}{2\varepsilon} + 2\varepsilon - \frac{\zeta_f}{\beta^2} \int_{t-\bar{\tau}}^{t} \|u(\theta)\|^2 d\theta.\end{aligned}$$

Using nonlinear damping to separate the terms and further bounding results in

$$\begin{aligned}\dot{V} \leq & -\left(\alpha_0 - \frac{1}{2}\right) e_0^T e_0 - \left(\alpha_1 - \frac{1}{2}\right) e_1^T e_1 \\ & - r^T \left(b_d k - \mathit{\Lambda}\right) r - S^T \left(\beta - \vartheta - \frac{\bar{\tau}\zeta_f}{\beta}\right) S \\ & - k_o y^T y + \frac{\left(\rho_1(\|z\|) + \rho_2(\|z\|)\right)^2 \|z\|^2}{2\zeta} \\ & + \frac{\left(\epsilon_1 + \epsilon_2\right)^2}{2\zeta} + 2\varepsilon - \frac{\zeta_f}{\beta^2} \int_{t-\bar{\tau}}^{t} \|u(\theta)\|^2 d\theta.\end{aligned}$$

Further, by splitting the integral term and using the Cauchy Schwarz inequality

$$\|e_I\|^2 \leq \bar{\tau} \int_{t-\bar{\tau}}^{t} \|u(\theta)\|^2 d\theta$$

the following term can be bounded as

$$-\frac{\zeta_f}{2\bar{\tau}\beta^2} \left(\bar{\tau} \int_{t-\bar{\tau}}^{t} \|u(\theta)\|^2 d\theta\right) \leq -\frac{\zeta_f}{2\bar{\tau}\beta^2} \|e_I\|^2.$$

This expression can be bounded as

$$\begin{aligned}\dot{V} \leq & -\left(K_{min} - \frac{\left(\rho_1(\|z\|) + \rho_2(\|z\|)\right)^2}{2\zeta}\right) \|z\|^2 \\ & - S^T \left(\beta - \vartheta - \frac{\bar{\tau}\zeta_f}{\beta}\right) S - k_o y^T y \\ & + \frac{\left(\epsilon_1 + \epsilon_2\right)^2}{2\zeta} + 2\varepsilon - \frac{\zeta_f}{2\beta^2} \int_{t-\bar{\tau}}^{t} \|u(\theta)\|^2 d\theta, \qquad (37)\end{aligned}$$

where K_{min} is defined as $K_{min} = \min\left\{\alpha_0 - \frac{1}{2}, \alpha_1 - \frac{1}{2}, \gamma_{min}\left\{b_d k - \mathit{\Lambda}\right\}, \frac{\zeta_f}{2\bar{\tau}\beta^2}\right\}$. Because

$$\begin{aligned}\int_{t-\bar{\tau}}^{t} \left(\int_{s}^{t} \|u(\theta)\|^2 d\theta\right) ds & \leq \bar{\tau} \sup_{s \in [t, t-\bar{\tau}]} \left[\int_{s}^{t} \|u(\theta)\|^2 d\theta\right] \\ & = \bar{\tau} \int_{t-\bar{\tau}}^{t} \|u(\theta)\|^2 d\theta,\end{aligned}$$

(37) can be rewritten as

$$\begin{aligned}\dot{V} \leq & -\left(K_{min} - \frac{\left(\rho_1(\|z\|) + \rho_2(\|z\|)\right)^2}{2\zeta}\right) \|z\|^2 \\ & - S^T \left(\beta - \vartheta - \frac{\bar{\tau}\zeta_f}{\beta}\right) S - k_o y^T y \\ & + \frac{\left(\epsilon_1 + \epsilon_2\right)^2}{2\zeta} + 2\varepsilon - \frac{1}{2\bar{\tau}} P, \qquad (38)\end{aligned}$$

Using the definitions of $x(t)$ and $z(t)$ this expression can be upper bounded as

$$\begin{aligned}\dot{V} \leq & -\bar{K}_{min} \|x\|^2 - \left(K_{min} - \frac{\left(\rho_1(\|z\|) + \rho_2(\|z\|)\right)^2}{2\zeta}\right) \|e_I\|^2 \\ & + \frac{\left(\epsilon_1 + \epsilon_2\right)^2}{2\gamma} + 2\varepsilon, \qquad (39)\end{aligned}$$

where $\bar{K}_{min}$ is defined as

$$\bar{K}_{min} = \min\left\{\begin{array}{c} K_{min} - \frac{\left(\rho_1(\|z\|) + \rho_2(\|z\|)\right)^2}{2\zeta}, \\ \beta - \vartheta - \frac{\bar{\tau}\zeta_f}{\beta},\ k_o,\ \frac{1}{2\bar{\tau}} \end{array}\right\}.$$

This expression can be further bounded if $K_{min} - \frac{\left(\rho_1(\|z\|) + \rho_2(\|z\|)\right)^2}{2\zeta} > 0$, which is true if the condition $\|z\|^2 < \bar{\rho}^{-2}(\sqrt{2K_{min}\zeta})$ is satisfied, where $\bar{\rho}$ is a positive monotonically increasing bounded function defined as $\bar{\rho} = \rho_1(\|z\|) + \rho_2(\|z\|)$. Considering (35), the definitions of x and z, and $P > \frac{\zeta_f}{\bar{\tau}\beta^2} e_I^2$, a set $\mathbb{S}$ can be defined as

$$\mathbb{S} \triangleq \left\{ x(t) \in \mathbb{R}^{9n+1} | \, \|x(0)\| < \sqrt{\frac{\lambda_1}{\lambda_2}\left(\min\left\{1, \frac{\zeta_f}{\bar{\tau}\beta^2}\right\} \bar{\rho}^{-2}\left(\sqrt{2\zeta K_{min}}\right) - \frac{\Upsilon}{\lambda_1}\right) - \frac{B\lambda_2}{\delta\lambda_1}} \right\}$$

where $B \in \mathbb{R}^+$ is a subsequently defined constant. In $\mathbb{S}$, $\bar{K}_{min}(\|z\|)$ is bounded by a constant $\delta \in \mathbb{R}^+$ as

$$\bar{K}_{min}(\|z\|) \geq \delta.$$

Adding and subtracting $\frac{\delta}{\lambda_2}\Upsilon$ to (39) and using (35), (39) becomes

$$\dot{V} \leq -\frac{\delta}{\lambda_2} V + B, \qquad (40)$$

where $B = \frac{\delta}{\lambda_2}\Upsilon + \frac{\left(\epsilon_1 + \epsilon_2\right)^2}{2\zeta} + 2\varepsilon$. (40) can be integrated with respect to time to obtain

$$V(x,t) \leq V(0) e^{-\frac{\delta}{\lambda_2} t} + \frac{B\lambda_2}{\delta}\left(1 - e^{-\frac{\delta}{\lambda_2} t}\right). \qquad (41)$$

Based on (41) and (35), the condition in Theorem 1 in the Appendix, (33), can be restated as

$$\|x(t)\| \leq \sqrt{\frac{\lambda_2}{\lambda_1}\left(\|x(0)\|^2 - \frac{B}{\delta}\right)} e^{-\frac{\delta}{2\lambda_2} t} + \sqrt{\frac{\lambda_2 B}{\lambda_1 \delta}}.$$

Using this expression, the definition of $x(t)$, and (11), an explicit bound on the tracking error $e(t)$ can be derived as

$$\|e(t)\| \leq (1 + \alpha_0)\left(\sqrt{\frac{\lambda_2}{\lambda_1}\left(\|y(0)\|^2 - \frac{B}{\delta}\right)} e^{-\frac{\delta}{2\lambda_2} t} + \sqrt{\frac{\lambda_2 B}{\lambda_1 \delta}}\right).$$

■
